# The alternative splicing program of differentiated smooth muscle cells involves concerted non-productive splicing of post-transcriptional regulators

**DOI:** 10.1093/nar/gkw560

**Published:** 2016-06-17

**Authors:** Miriam Llorian, Clare Gooding, Nicolas Bellora, Martina Hallegger, Adrian Buckroyd, Xiao Wang, Dipen Rajgor, Melis Kayikci, Jack Feltham, Jernej Ule, Eduardo Eyras, Christopher W.J. Smith

**Affiliations:** 1 Department of Biochemistry, University of Cambridge, Tennis Court Road, Cambridge, CB2 1QW, UK; 2 Computational Genomics, Universitat Pompeu Fabra, E08003 Barcelona, Spain; 3 INIBIOMA, CONICET-UNComahue, Bariloche 8400 Río Negro, Argentina; 4 Department of Molecular Neuroscience, UCL Institute of Neurology, Queen Square, London, WC1N 3BG, UK; 5 MRC-Laboratory of Molecular Biology, Francis Crick Avenue, Cambridge CB2 0QH, UK; 6 Catalan Institute for Research and Advanced Studies (ICREA), E08010 Barcelona, Spain

## Abstract

Alternative splicing (AS) is a key component of gene expression programs that drive cellular differentiation. Smooth muscle cells (SMCs) are important in the function of a number of physiological systems; however, investigation of SMC AS has been restricted to a handful of events. We profiled transcriptome changes in mouse de-differentiating SMCs and observed changes in hundreds of AS events. Exons included in differentiated cells were characterized by particularly weak splice sites and by upstream binding sites for Polypyrimidine Tract Binding protein (PTBP1). Consistent with this, knockdown experiments showed that that PTBP1 represses many smooth muscle specific exons. We also observed coordinated splicing changes predicted to downregulate the expression of core components of U1 and U2 snRNPs, splicing regulators and other post-transcriptional factors in differentiated cells. The levels of cognate proteins were lower or similar in differentiated compared to undifferentiated cells. However, levels of snRNAs did not follow the expression of splicing proteins, and in the case of U1 snRNP we saw reciprocal changes in the levels of U1 snRNA and U1 snRNP proteins. Our results suggest that the AS program in differentiated SMCs is orchestrated by the combined influence of auxiliary RNA binding proteins, such as PTBP1, along with altered activity and stoichiometry of the core splicing machinery.

## INTRODUCTION

Alternative splicing (AS) is a key contributor to remodeling the transcriptomes of cells during development and differentiation. Numerous analyses have indicated the functional importance of AS, and highlighted the fact that AS and transcriptional control tend to operate on different sets of genes (1,2). Much has been learned about the *cis* and *trans*-acting components of ‘splicing codes’ that give rise to regulated splicing programs characteristic of various adult tissues. Cell-specific splicing regulatory mechanisms have been intensely investigated in many cell types using both global profiling and computational techniques, as well as detailed molecular analysis of individual model splicing events (3). Splicing programs have been well characterized in cardiac and skeletal muscle (reviewed in (4)). By comparison, despite their biomedical importance, knowledge of the regulated splicing programs of smooth muscle cells (SMCs) is more rudimentary.

SMCs are important in a number of physiological systems and organs, including the cardiovascular system. The hallmark property of differentiated SMCs is the ability to contract, but contractile SMCs themselves show a range of phenotypes allowing prolonged tonic contraction in vascular smooth muscle or rapid phasic contraction in tissues such as bladder (5). Another distinctive characteristic, in contrast with terminally differentiated striated muscle cells, is that SMCs exhibit phenotypic plasticity (6). Vascular SMCs are able to modulate their phenotype along a continuum between a contractile phenotype, characteristic of healthy blood vessels and a more proliferative ‘synthetic’ phenotype, so-named for the enhanced synthesis and secretion of extracellular matrix (ECM) components (5–9). Synthetic phenotype cells are found in a number of pathological situations such as atherosclerosis and arterial injury (8). Phenotypic modulation can be modeled in cultured rat aortic SMCs, and much is known about the changes in gene expression levels and the underlying transcription control networks (5,8). Likewise, the contribution of miRNA regulation to SMC phenotype has been well documented (10). In contrast, the contribution of AS to shaping the SMC transcriptome has so far been restricted to detailed analysis of a small number of model AS events (ASE) that are regulated in contractile SMCs compared to synthetic cells or other tissues (e.g. (11–16)), or that are regulated between contractile SMCs from tonic and phasic smooth muscle tissues (17–19). While interesting mechanistic and functional insights have been gained from these studies, it is difficult to draw general inferences about the regulatory principles of a smooth muscle splicing program. For example, polypyrimidine tract binding protein (PTBP1) regulates mutually exclusive exon selection in both the *Tpm1* and *Actn1* genes with smooth muscle specificity. However, it acts in ‘opposite’ directions, repressing the smooth muscle-specific exon of *Actn1* (13–15,20), but in *Tpm1* repressing the ‘default’ exon 3 thereby facilitating exon 2 inclusion in SMCs (21–23).

Here, we used mouse exon-junction (MJAY) arrays (24) to gain insights into both the global contribution of ASE in re-shaping the transcriptome of dedifferentiating SMCs, and into the underlying regulatory mechanisms. We observed numerous changes in both AS and transcript levels, which affected different sets of genes. Cassette exons (CEs) used in differentiated cells were characterized by particularly weak splice sites, and by the presence of PTBP1 binding sites in the upstream intron, associated with repression of the exons by PTBP1 in proliferative cells. Finally, we observed a concerted set of non-productive splicing events within the genes for snRNP proteins, other splicing factors and other post-transcriptional regulators. These splicing events, which included intron retention (IR), ‘poison’ CE (i.e. CEs that introduce premature termination codons (PTC)) inclusion and alternative polyadenylation, were all predicted to lead to lower expression of the cognate proteins in differentiated SMCs. In contrast, levels of spliceosomal snRNAs, particularly U1, were higher in differentiated compared to proliferative cells, suggesting heterogenous snRNP composition in these cells. Taken together, our results suggest that the regulation of the AS program in SMCs is regulated both by auxiliary RNA binding proteins and by altered levels of core splicing factors and snRNP composition.

## MATERIALS AND METHODS

### Mouse primary cells and tissue samples

Smooth muscle tissue from mouse aorta and bladder was isolated from 10–20 week old C57BL/6 mice. Pools of five aorta or bladder were used to harvest RNA from differentiated tissue by chopping the tissue into small pieces and placing in RNAlater (Qiagen) before subsequently extracting RNA with the Ribopure kit (Ambion). Single cell cultures were produced from Ultra-Turrax T8 homogenized tissues using established protocols for mouse aorta SMCs (25). Briefly, five aortas or bladders were incubated with shaking in 3–5 ml of 1 mg/ml collagenase (Sigma) and 3 mg/ml elastase (Worthington Biochemical Corporation) at 37 °C for ∼1 h. Cells were washed in phosphate buffered saline (PBS) and larger aggregates removed with a cell strainer. Cells were counted and either resuspended in 4% sodium dodecyl sulphate, 125 mM Tris pH6.8, 1 mM DTT, 10% glycerol for protein lysates or plated at 4 × 10^5^ ml^−1^ in Dulbecco's modified Eagle's medium (DMEM), 10% fetal bovine serum (FBS), 2 mM Glutamine, 1 mM Sodium Pyruvate, 1× penicillin/streptomycin. Medium was changed on day 2 and the cells split 1:2 if necessary before harvesting on day 7 or when the cells had grown to ∼80% confluent.

### Arrays and analysis

RNA from three biological replicates each of aorta medial layer, aorta SMCs cultured for 7 days but not passaged, bladder smooth muscle and cultured SMCs was isolated using the Ribopure kit (Ambion). Total RNA was used to prepare target for hybridization to Affymetrix Mouse Exon-Junction Array (MJAY) (26,27). The microarray data was analyzed using ASPIRE 3.0 (Analysis of SPlicing Isoform REciprocity, available at https://github.com/pandora2017/ASPIRE) which analyses signal in reciprocal probe-sets to monitor changes in ASE using the dIrank statistic, which is a modified *t*-test to sort the data based on the significance and direction of change. We routinely applied a threshold of |dIrank|≥1 for significant events (28,29). Venn diagrams were generated using BioVenn (30)

### RT-qPCR

cDNA was prepared using 200 ng total RNA extracted from differentiated or proliferative mouse samples, using 100 ng/μl oligo dT and Superscript II (Life technologies) following manufacturer's instructions. Changes in isoform expression were measured by real time polymerase chain reaction (PCR). Primers were designed by Primer3Plus (see Supplementary Data File 12 for primers). Exon–exon junction primers were designed specifically to detect one single isoform. Primer performance and specificity was monitored by unique melting profile and presence of a single band on agarose gel electrophoresis. QPCR reactions were carried out using 10 ng cDNA, 400 nM of each primer with 1× SYBR^®^ Green JumpStart^™^ *Taq* ReadyMix^™^ from Sigma. Reactions were run in a Rotor-Gene Q instrument (QIAGEN), following a three-step protocol. QPCR analysis was carried out using the Comparative Quantitation tool within the Rotor-Gene Q Series Software 1.7. Relative expression values were normalized against the geometric mean of 5 genes predicted not to change by the microarray analysis (Alkhb3, Hgsnat, Atp6v1e1, Bmpr2, Pol2rb). Reactions were carried out on biological triplicates and changes were considered significant at *P* < 0.05 (*), < 0.01 (**) and < 0.001 (***) by two-tailed unpaired *t*-test.

For conventional RT-PCR 50 ng cDNA reaction was used as template with 400 nM primers, 3 mM MgCl_2_, 200 μM dNTPs and JumpStart™ *Taq* DNA Polymerase (SIGMA). PCR reactions were carried out at 60°C following manufacturer's instructions. PCR products were resolved in a QIAxcel Advanced System (QIAGEN) and percentage of isoform inclusion measured with QIAxcel ScreenGel software. Images shown are computer generated from electropherograms. Values shown are mean ± STDEV.

### Western blot

Standard protocols for Laemmli gels and western blotting were used, loading 50 μg of total protein for both single cells (D) and day 5–7 (P) cell lysates. Antibodies for western blotting from Abcam were U1A (ab55751), Sf3b1 (ab39578), Sf3b3 (ab96683), Srsf7 (ab138022), Tra2 β (ab66901), Rbm3 (ab134946), hnPNPL (ab6106) and Tubulin (ab6160). Antibodies from Santa Cruz Biotechnology were U1C (sc-101549), U2A’ (sc-393803), Srsf6 (sc-34198), Rbp1 (sc-56767) and Gapdh (sc-25778). The remaining antibodies were U170K/Snrnp70 Synaptic Systems (203 011), Srsf1 Invitrogen (32-4500), PTBP1 Invitrogen (324800), PTBP2 (kindly provided by Michele Solimena), QKI (UC Davis/NIH NeuroMab Facility clone N147/6), PTBP3 provided by Elisa Monzon-Casanova, Acta2 Dako (M0851) and Myh-11 Sigma (M7786).

### PAC1 cell culture

Rat PAC1 pulmonary artery SMCs (31) were grown in DMEM supplemented with Glutamax (Invitrogen) and 10% FBS. To encourage the cells toward a more differentiated state they were sub-cultured at a 1:10 dilution once per week and for the more proliferative state they were sub-cultured 1:20 twice per week. For siRNA transfection cells were seeded at 10^5^ cells per well and 24 h later transfected with 120 pmols of siRNA and oligofectamine. A total of 90 pmols of P3 siRNA were used against PTPB1 and 30 pmols of N1 siRNA against PTBP2 (22) and 20 pmol Q1 for QKI (32). Cells were transfected 24 h later using lipofectamine 2000 with the same concentration of siRNAs. Protein and RNA were harvested 48 h after the second hit. Knockdown efficiency was measured by running 10 μg of total cell lysate on a sodium dodecyl sulphate-polyacrylamide gel electrophoresis gel and western blot. QKI knockdown was also assessed by qRT-PCR. siRNA knockdown experiments were done four times, and each time *n* = 3 at least for each of the siRNAs. For plasmid transfections, 2 × 10^5^ cells/well were seeded in 6-well tissue culture plates 24 h before transfection with 5 μl SuperFect (QIAGEN) and 2 μg DNA with minigene:effector plasmids in a 1:4 ratio. RNA and protein were harvested 48 h later. When plasmid transfection was combined with RNAi, the plasmid was transfected 5 h before the second siRNA treatment. For cycloheximide treatments, 2 × 10^5^ cells were seeded on 6-well plates and 24 h later, 3 wells were treated with 100 mg/ml cycloheximide for 8 h and 3 wells were treated with equal volume of DMSO. Cells were harvested in TRI reagent (Sigma) and RNA was extracted following manufacturer's instructions. Cycloheximide treatments were done on three independent occasions. TG003 (T5575, SIGMA) was added to PAC1 cells for 2 h at a concentration of 20 μM. After treatment cells were harvested in TRI reagent following manufacturer's instructions and changes in splicing monitored by RT-PCR and QIAxcel. Nuclear cytoplasmic fractions from PAC1 cells were prepared using NE-PER™ Nuclear and Cytoplasmic Extraction Reagents (Thermo). From the resulting fractions RNA was extracted using TRI-reagent and RT-PCR was carried out as explained above.

### Immuno-staining and RNA fluorescence *in situ* hybridization (FISH)

PAC1 cells grown on coverslips were fixed for 10 min at room temperature (RT) in 4% paraformaldehyde (PFA), rinsed with PBS and permeabilized with 0.5% NP-40 for 2 min. Coverslips were again rinsed with PBS and incubated with blocking solution (1% BSA) for 1 h at RT. Primary antibodies were diluted in blocking solution and applied to coverslips for 1 h at RT followed by three rinses in PBS. Primary antibodies were: Rabbit anti-Snrnp70 (Milipore 06–1297) diluted at 1:100, rat anti-U1C (Santa Cruz sc-101549) at 1:100 and mouse anti-smActin Dako M0851 at 1:1000. Coverslips were incubated with secondary antibodies, diluted 1:500 in blocking buffer, conjugated to Cy2 or Cy3 (Jackson Laboratory) for 1 h at RT. The coverslips were next stained with DAPI and washed with PBS before being mounted in ProLong Gold antifade reagent (Invitrogen).

For RNA fluorescence *in situ* hybridization (FISH) PAC1 cells grown on coverslips were fixed for 10 min at RT in 4% PFA, dehydrated by serial incubation with 50% EtoH for 2 min, 70% EtOH for 2 min and 100% EtOH for 2 min. Coverslips were stored at −20°C in 100% EtOH until use. Cells were rehydrated by incubating with 70% EtOH for 2 min and 50% EtOH for 2 min. The U1 snRNA probe (Cy3-5′GCCAGGUAAGUAU3′) was used at 2 ng/μl in 100% formamide and heat denatured at 80° C for 10 min before adding 1–5 μl of 2× hybridization buffer (4× SSC, 20% dextran sulphate, 0.4% BSA in DEPC-water) followed by 1 μl bakers yeast tRNA (1 ng/μl in formamide) and 3 μl of formamide. Coverslips were spotted onto the hybrization mix and hybrizided at 37°C overnight. Cells were washed in in 2× SSC 50% formamide at 37°C for 15 min, 2× SSC at 37°C for 15 min, 1× SSC at 37° C for 15 min and then equilibrated in 4× SSC for 2 min at RT. Coverslips were stained with DAPI and washed twice with 1× SSC before being mounted onto slides. Immunofluorescence and RNA FISH images were acquired using a Zeiss wide-field microscope attached to a Zeiss Axiocam MRm camera under a 40×/1.4 NA oil objective.

### Bioinformatic analyses

Analysis of functional gene categories regulated by AS or transcription was carried out using the statistical over-representation tool of PANTHER v10.0 (33) with Bonferroni correction for multiple testing, which outputs both over- and under-represented terms. We compiled reference sets of genes (aorta, 6347; bladder, 6941) expressed above a threshold level (ASPIRE TA ≥ 500) in either D and/or P cells. For AS, a threshold of |dIrank| > 5 was used, and for transcript levels a 3-fold change threshold. For analysis of CE properties we compiled sets of exons upregulated in differentiated or proliferative cells (dIrank > 1 or < -1 respectively) of both aorta and bladder, with an additional cut-off of a predicted change in percent inclusion of >20% (248 differentiated exons and 106 proliferative exons). As a control unregulated set we used 2633 well annotated CEs with predicted change of percent inclusion of <5% in both tissues. To investigate the properties of regulated retained introns we compiled a set of 123 introns that were more retained in both aorta and bladder differentiated cells (dIrank > 1 and dI > 20). These were compared with a control set of annotated retained introns that were not regulated in aorta or bladder (dIrank < 1 and dI <10), and with a set of annotated constitutively spliced introns (see below).

Exonic and intronic sequences were retrieved from the mouse assembly (NCBI37/mm9). The set of constitutive introns (CIs) were defined from genes with alternative events found in the array when intronic regions satisfying the following criteria: do not overlap nor flank with events, neither with UCSC alternative events and present in all annotated Ensembl isoforms.

Two-sided Mann–Whitney test were used to compare the sequence properties between datasets. All statistical tests were performed and graphics were generated with R (34).

Motif enrichments were calculated using 100 bp of the flanking exons and the complete sequence of the CEs. For introns we used maximum intronic flanks of 250 nt, removing 9 nt at donor side and 30 nt at the acceptor side to avoid branch point (BP), splice site (SS) and polypyrimidine tract (PPT) signals, (35), retrieving introns with a minimum length of 60 nt. We assess the enrichment of 5mers and RNA compete motif matches (36) with the procedure described in Coelho *et al*. (26).

Statistically over-represented motifs were selected based on the Benjamini and Hochberg false discovery rate multiple test corrected p-value (BH-FDR < 0.05). Additional scripts were written in PHP and Awk, and sequence logos generated with seqlogo (37).

## RESULTS

**Figure 1.**
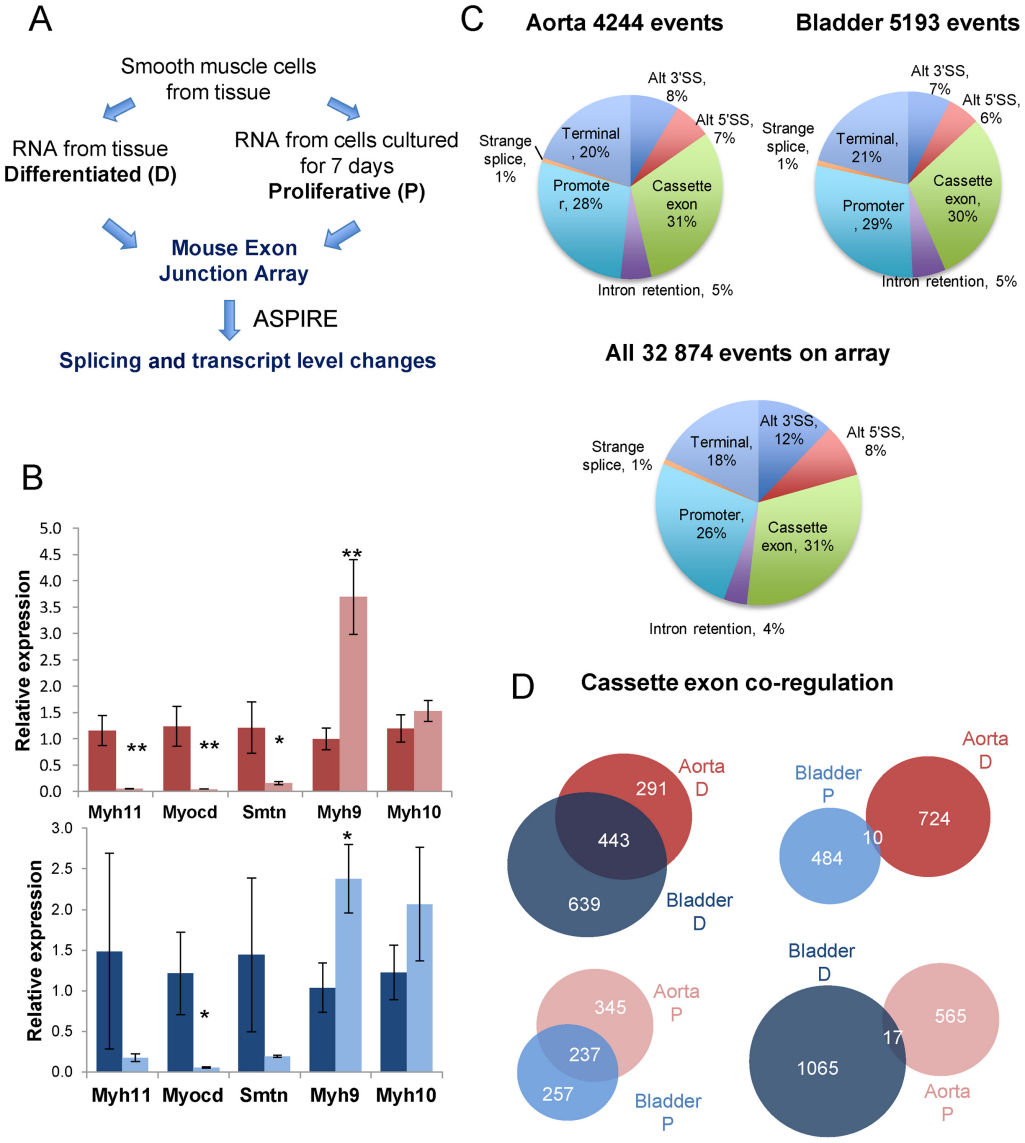
Splice sensitive array profiling of dedifferentiating smooth muscle cells (SMCs). (**A**) Overall experimental design. RNA was isolated from differentiated contractile SMCs of mouse aorta and bladder, and from SMCs that had been cultured for 7 days. RNA was used to prepare target for hybridization to splice-sensitive microarrays, followed by prediction of transcript level and splicing changes by ASPIRE. (**B**) qRT-PCR analysis of changes in marker gene expression between differentiated (dark) and proliferative (light) aorta (red) and bladder (blue). (**C**) Distribution of regulated ASE types between differentiated and proliferative aorta (top left) and bladder (top right) SMCs, and of all ASEs represented on the array (bottom). For this analysis events annotated on the array as ‘promoter’ and ‘bleeding promoter’ were combined, as were ‘terminal’ and ‘bleeding terminal’. (**D**) Venn diagram comparing direction of changes in CE splicing in aorta and bladder SMCs. The vast majority of CEs are coregulated in the two tissues.

**Figure 2.**
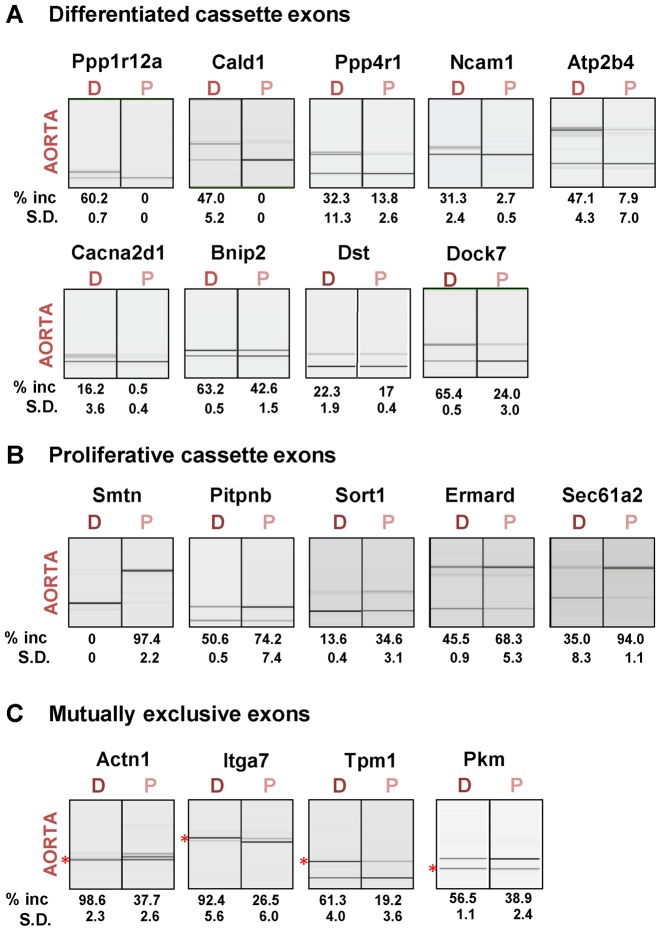
Validation of cassette and mutually exclusive splicing events. Selected cassette exons (CEs) with increased inclusion in differentiated (**A**) or proliferative cells (**B**) and mutually exclusive exon events (**C**) were validated by RT-PCR for both aorta and bladder. Values shown are mean ± sd (*n* = 3) of percentage of exon inclusion. In panel C, the % inclusion of the ‘differentiated exon is indicated by the red asterisk; for Actn1 the differentiated exon is smaller; for Itga7 and Tpm1 the differentiated exon amplicon is larger. For every primer pair, a no RT control and a no template control were run in parallel; no signal was detected in any of the reactions (not shown). D = differentiated, *P* = proliferative phenotype.

We used mouse aorta and bladder as sources of SMCs. These two tissues showed the strongest signal in transgenic mice carrying a splice-sensitive fluorescent reporter based on *Tpm1* SMC-specific AS (38), and also provide contrasting examples of tonic and phasic smooth muscle tissues (5). Triplicate RNA samples were purified from the intact medial layer of mouse aortas and the detrusor muscle of bladder, and from SMCs that had been isolated from the two tissues and cultured for 4–7 days without further passaging. We subsequently refer to the tissue-derived samples as differentiated (D) and the cultured cells as proliferative (P) SMCs. Preliminary analysis indicated that known ASE in the *Tpm1* (39) *Actn1* (15,40), *Myocd* (16,41) and *Vcl* genes (42) were all regulated between the differentiated and proliferating SMCs, as expected (Figure 2 and data not shown). RNA was used to prepare target for hybridization to MJAY exon-junction arrays featuring probe-sets for all Refseq and Ensembl annotated exons and exon-exon junctions. Data analysis using the ASPIRE3 pipeline (29), produced sets of predictions for changes in expression levels and in AS during dedifferentiation of SMCs from both tissues (Figure 1A; Supplementary Data Files 1 and 2).

The array analysis indicated the expected changes in expression levels of known SMC transcriptional markers during phenotypic modulation. Reverse transcription followed by qPCR confirmed that in aorta Myocardin (Myocd) was downregulated by 31-fold, smoothelin (Smtn) by 7.6-fold and smooth muscle myosin heavy chain (Myh11) by 23-fold (Figure 1B), while non-muscle myosin heavy chain 9 (Myh9) was upregulated by 3.7-fold. Similar changes were observed in bladder (Figure 1B), although only the changes in Myocd and Myh9 were significant.

A total of 4246 ASE in aorta and 5193 in bladder were predicted to change significantly (Figure 1C and Supplementary Data File 1). The distribution of regulated event types was similar to that of all annotated events represented on the arrays (Figure 1C), consistent with a regulated program involving AS, alternative promoter selection and 3′ end selection. In both tissues CE, at ∼30%, were the largest class of regulated ASE (Figure 1C). In aorta, 734 CEs were more included in differentiated and 582 were more included in proliferative cultured cells. Similarly in bladder 1082 CEs were upregulated in differentiated, compared with 494 that were more included in proliferative SMCs. Direct comparison of splicing patterns between differentiated aorta and differentiated bladder SMCs (Supplementary Data File 1) showed about half the number of splicing differences, as compared to the differentiated to proliferative comparisons (2184 ASEs, including 702 CEs). Comparing ASEs that were regulated between differentiated and proliferative cells in both tissues, the vast majority were regulated in the same direction. For example, of the 734 CEs upregulated in differentiated aorta, 443 (60%) were regulated in the same direction in bladder, while only 10 (1.4%) were regulated oppositely (Figure 1D). Likewise, of the 582 proliferative aorta exons, 237 (41%) were also upregulated in proliferative bladder and only 17 (2.9%) in differentiated bladder SMCs. In contrast, comparison with published data for mouse skeletal muscle C2C12 cells in proliferating myoblast and terminally differentiated myotube states (24) showed smaller overlaps with no favored direction of overlap. For example, 3.4% of aorta differentiated exons were also upregulated in differentiated C2C12 myotubes, while 4.0% were upregulated in proliferating myoblasts. Likewise, 2.4% of aorta proliferative exons were also upregulated in myoblasts and 4.6% in myotubes. Thus, we have identified a large set of co-regulated exons that are regulated specifically in SMCs of diverse types.

The regulated ASEs include some that have previously been analyzed experimentally including mutually exclusive exon pairs in the *Tpm1* (11,12,43) and *Actn1* genes (13–15) and CE 23 in the myosin phosphatase regulatory subunit gene Pppr1r12a (17). A selection of validated exons are shown in Figure 2 and Supplementary Figure S2, including exons with increased inclusion in differentiated (Figure 2A) or proliferative (Figure 2B) SMCs, as well as mutually exclusive exon pairs (Figure 2C). CEs in Ppp1r12a, Atp2b4 and Cacna2d1 also showed significantly higher inclusion in differentiated bladder compared to aorta (Figure 2 and Supplementary Figure S2), consistent with ASPIRE comparison of aorta and bladder tissue samples (Supplementary Data File 1), and with previous analyses of Ppp1r12a exon 23 (17).

Over-represented functional terms associated with genes affected by AS, or transcript-level regulation were examined using PANTHER (33) (Supplementary Data File 3). The following discussion refers to aorta, but similar observations were made for bladder. Few significantly over-represented terms for genes affected by AS were observed. However, when a higher threshold was applied (|dIrank| >5), the top enriched term was ‘actin family cytoskeleton’ (*P* = 4.04E-09) for all regulated ASEs, and ‘non-motor actin binding protein’ for CEs (*P* = 2.64E-03). In contrast, the most significantly enriched protein function terms for genes upregulated by > 3-fold in differentiated compared to proliferative cells were receptor (*P* = 4.0E-07), ion channel (*P* = 4.35E-7), and voltage gated ion channel (*P* = 1.66E-05), which included a series of K^+^, Ca^2+^ and Na^+^ channels important for the excitable contractile state. Genes transcriptionally upregulated in proliferative samples showed over-representation of signaling molecule (*P* = 6.09E-04) and oxygenase (*P* = 1.17E-03). Strikingly, ‘RNA binding proteins’ were significantly under-represented among genes upregulated in either differentiated (*P* = 3.63E-08) or proliferative samples (*P* = 1.19E-06). Consistent with this, the array data predicted <2-fold changes in expression levels of individual splicing regulators such as Ptbp1, Mbnl1, Mbnl2, Srsf1, Srsf2, Srsf6 and Sfrs10/Tra2β between differentiated and proliferative aorta SMCs. This was confirmed by qRT-PCR for Srsf1, Srsf6, Sfsr10 (Tra2β), Cugbp1, Cugbp2, HnrnpL, Ptbp2, Ptbp3, Mbnl1 and Mbnl2 (Supplementary Figure S1). Levels of Ptbp1 were observed to increase by 2.03-fold (*P* = 0.003) between differentiated and proliferative aorta, and a similar increase was also observed at the protein level (see below). However, the overall modest transcriptional regulation of RNA binding proteins between differentiated and proliferative SMCs is striking in view of the extensive program of AS, and suggests that regulation of this program might be orchestrated at levels other than transcription or turnover of the mRNAs for regulatory RNA binding proteins.

### Properties of smooth muscle cassette exons

**Figure 3.**
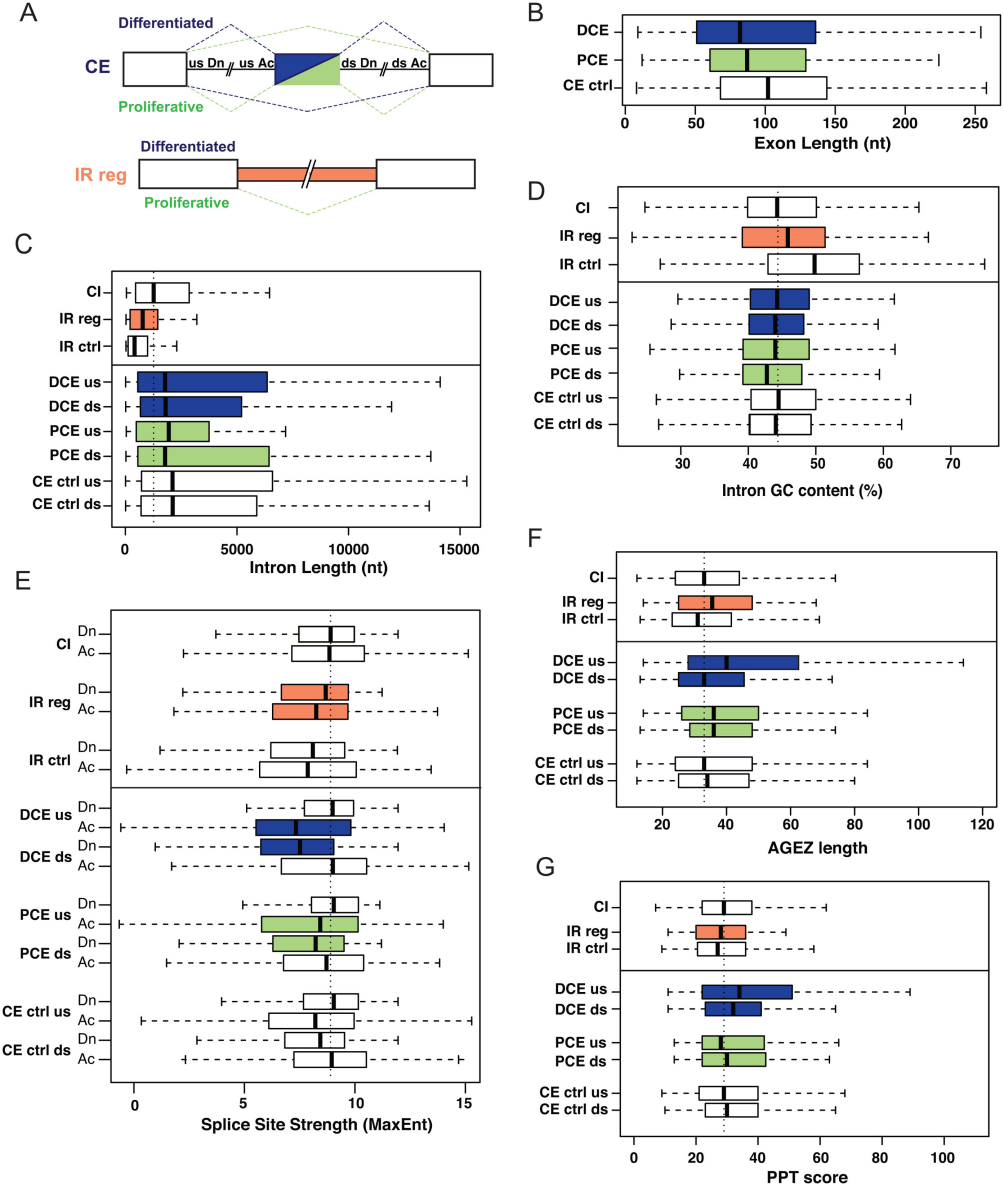
Properties of SMC regulated CEs and retained introns. (**A**) Schematic of the experimental sets of exons and introns. Top: CEs. Differentiated exons represented in dark blue, proliferative exons in green. Bottom: regulated intron retention (IR reg), more retained in differentiated cells. (**B**) Length distributions of CEs. (**C**) Length distributions of introns. (**D**) Intron GC content (%). (**E**) Splice site strength (Maximum Entropy). Dn = donor or 5′ splice site, Ac—acceptor or 3′ splice site. (**F**) AG dinucleotide exclusion zone (AGEZ) (**G**) Polypyrimidine tract score associated with best predicted branch point. Datasets analyzed in the figure: regulated CEs in proliferative (PCE) and differentiated (DCE) cells, control CEs unregulated in SMCs (CE ctrl), constitutive introns (CIs), retained introns regulated in SMCs (IR reg), non-regulated retained introns (IR ctrl). DCE us—intron upstream of differentiated CE, DCE ds—intron downstream of DCE, PCE us and PCE ds—introns upstream and downstream respectively of PCE, CE ctrl us and ds—introns upstream and downstream respectively of control CEs unregulated in SMCs. Statistical tests described in ‘Materials and Methods’ section, and significant differences are described in accompanying text. The vertical dashed line in each panel represents the median value from the CI set.

We next analyzed properties of the regulated CEs, focusing on events that are co-regulated in both smooth muscle tissues and that therefore represent a core smooth muscle AS program (Figure 3A). To this end, we collated sets of exons that are upregulated in differentiated (443 exons, Figure 1C) or in proliferative (237 exons, Figure 1C) cells. We applied an additional cut-off of a predicted change in percent inclusion of >20% to generate sets of exons that are robustly regulated in both tissues (248 differentiated exons and 106 proliferative exons). As a control set we used 2633 well-annotated CEs that were not regulated in either tissue.

Differentiated CEs (DCEs) were shorter than proliferative (PCE) or control CEs (median sizes 82, 87 and 102 nt respectively, DCE versus control CE, *P* = 2.02 × 10^−5^, Figure 3B). In contrast the size of flanking introns (Figure 3C) and their GC content (Figure 3D) did not differ significantly between the different CE sets. As expected for CEs (44), 5′ and 3′ splice site scores for all three groups of CE (DCE, PCE and CE ctrl) were lower than for their flanking constitutive exons (Figure 3E: compare ‘us Dn’ versus ‘ds Dn’, and ‘us Ac’ with ‘ds Ac’ for each set of CEs). However, the 5′ splice sites of DCEs were significantly weaker than those of the control CEs (*P* = 7.4 × 10^−5^, Figure 3E: DCE ds Dn versus CE ctrl ds Dn). Likewise, the 3′ splice sites of DCEs were weaker than those of control CE (*P* = 0.03, Figure 3E, DCE us Ac versus CE ctrl us Ac). The 3′ splice site scores are based upon 20 nt of intron sequence upstream of the exon (45), but branch points and their associated polypyrimidine tracts can be located much further upstream (46). We therefore used SVM-BP finder (47) to analyze the strengths of predicted branch points, their associated pyrimidine tracts and the size of the AG-dinucleotide exclusion zone (AGEZ), which is indicative of branch point location (46,47). DCE's had significantly longer AGEZ than their downstream constitutive exons (*P* = 0.001), or control CE (*P* = 10^−4^) (Figure 3F), and their polypyrimidine tracts (Figure 3G) were significantly stronger than control CEs (*P* = 0.0047). However, neither the branch point scores (*P* = 0.09, Supplementary Figure 3), nor the distance between the predicted branch point and 3′ ss (*P* = 0.08, Supplementary Figure S3C) differed significantly between differentiated and control CE. The CEs used in differentiated SMCs therefore share a number of properties that distinguish them from CEs in general, including a shorter size, weaker 5′ and 3′ splice sites, but stronger pyrimidine tracts and larger AGEZs.

**Figure 4.**
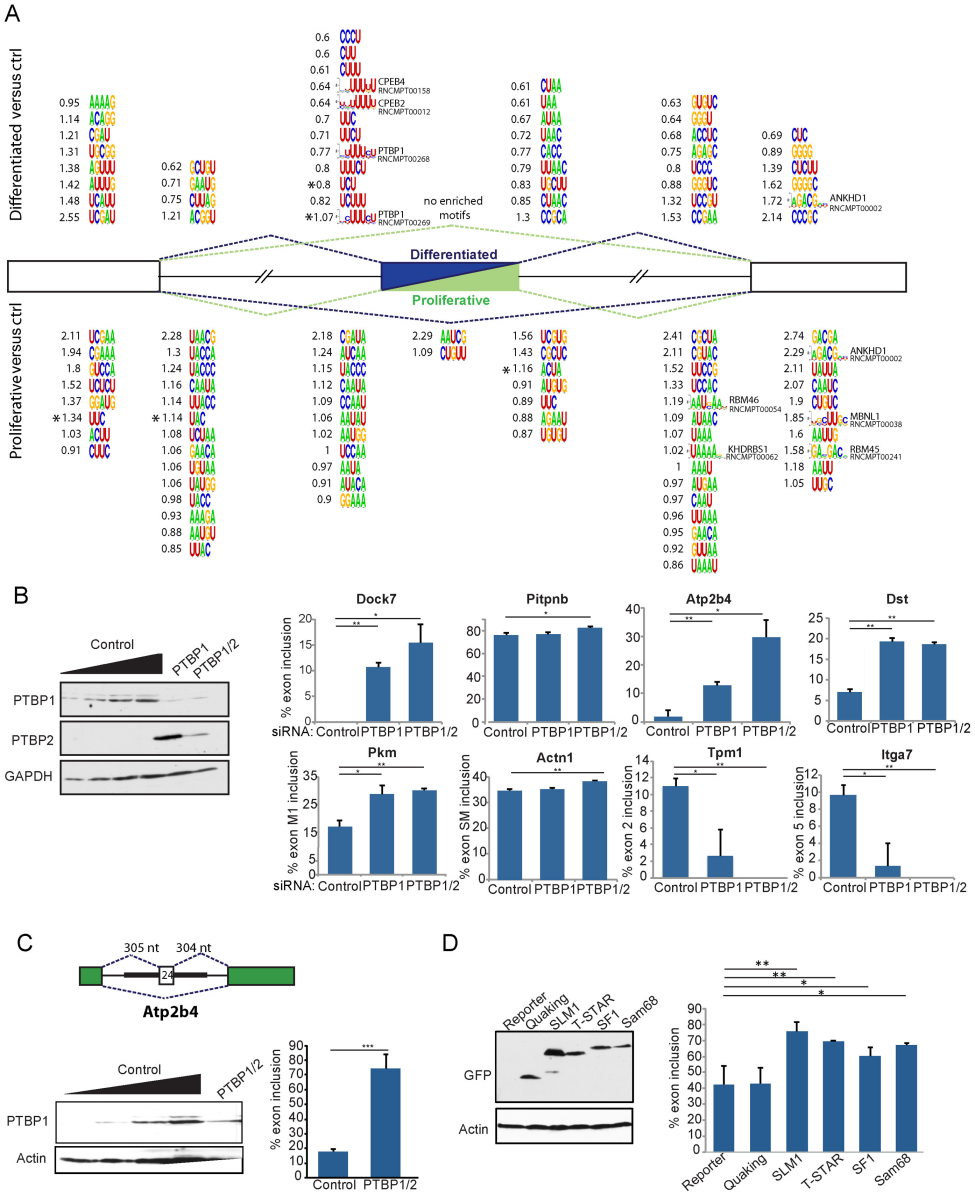
Sequence motif enrichments reveal a role for PTB in regulating SMC CEs. (**A**) Motif enrichment analysis. The diagram indicates k-mer and RNA-compete motifs enriched in the indicated regions associated with differentiated (top) or proliferative (bottom) exons. All motifs were significantly enriched (*P* < 0.01). The five motifs marked with an asterisk also passed a FDR test < 0.05. Numbers adjacent to motifs indicate log_2_ fold enrichment. (**B**) PTBP1 and PTBP2 were knockdown in rat PAC1 cells and its effects assessed in SMC CE from Figure 2. (**C**) Effect of PTBP1/2 knockdown in rat PAC1 cells on Atp2b4 minigene, containing the regulated exon with 225 nt of upstream intron and 275 nt of downstream intron, cloned into an GFP exon trapping vector. (**D**) Effect of overexpression of STAR family proteins on Atp2b4 minigene in rat PAC1 cells. Histograms show mean and standard deviation of the mean of at least three samples. Statistically significance was calculated using Student's *t*-test, and is shown *P < 0.05, **P < 0.01, ***P < 0.001.

We next analyzed the differentiated and proliferative CEs for enrichment or depletion of 3–5 mer sequence motifs. To avoid spurious cases of enrichment based upon long repeat elements in a small number of introns, we carried out the analyses by counting the cases where k-mer frequency is above a specified threshold (see ‘Materials and Methods’ section). We applied this approach for enrichment of k-mers (3–5 nt) as well as RNA-compete motifs corresponding to >200 RNA binding proteins (36). A moderate number of enriched k-mers and RNA-compete motifs (*P* < 0.01) were found in the seven transcript locations associated with DCEs (55 motifs) and PCEs (77 motifs) (Figure 4A). However, only two motifs associated with differentiated exons, and three with proliferative exons passed an FDR < 0.05 test (marked with asterisks in Figure 4A). The most obvious enrichment was the presence of multiple mixed C/U k-mers corresponding to PTBP1 binding sites (e.g. UCU, *P* = 5 × 10^−4^, FDR 0.03), as well as a PTBP1 RNA-compete motif (*P* = 5.8 × 10^−6^, FDR 10^−4^) on the upstream side of differentiated CEs, a location consistent with repression by PTBP1 (27,48). In contrast, such motifs were not associated with either flank of proliferative CEs. This suggests that PTBP proteins act commonly as repressors of smooth muscle specific exons, in non-contractile cells. No motifs were enriched within differentiated CEs and only two within proliferative CEs (Figure 4A). The only other motifs that passed a FDR test were UUC (*P* = 10^−4^, FDR 0.0064) in the constitutive exon upstream of proliferative exons, UAC (*P* = 6.1 × 10^−4^, FDR 0.039) along with related motifs (UUACC, UACC, UUAC, UACCC, UACCA) in the intron adjacent to the upstream constitutive exon and ACUA (*P* = 4 × 10^−4^, FDR 0.038) immediately downstream of proliferative exons. Other groups of similar motifs suggest possible roles for known splicing regulators. For example, a series of UAA containing motifs, including UUAAC (*P* = 0.0027), CUAAC (*P* = 0.0041) and ACUAA (*P* = 0.019) were all enriched on the downstream side of differentiated exons. These motifs all resemble the binding sites for STAR proteins such as Quaking, Sam68 and TSTAR (49).

To test for the potential role of PTBP proteins in regulating SMC ASEs, we knocked down PTBP1 and PTBP2 in rat PAC1 SMCs (Figure 4B). As in other cell types, knockdown of PTBP1 led to upregulation of PTBP2, but the elevated PTBP2 could be reduced by double PTBP1/PTBP2 knockdown. We analyzed the effects on 23 ASEs, including all of those shown in Figure 2, and 5 additional DCEs in the Srsf7, Srsf6, Sf3b3, Sfrs10 (Tra2β) and Myocd genes. The splicing patterns in the PAC1 cells resembled those of the more proliferative mouse SMCs (compare Figures 4B and 2). Eight ASEs were found to be affected significantly (Figure 4B), five of which are DCEs upregulated by PTBP1 or PTBP1/2 knockdown (Dock7, Atp2b4, Dst, Pkm, Actn1). This is consistent with PTBP1 repressing these DCEs via binding sites upstream of exons (Figure 4A). Tpm1 mutually exclusive exons 2 and 3 are an atypical PTBP1 regulated event, in which PTBP1 represses the non-muscle exon 3 (21,50). Itga7 mutually exclusive exons 5 and 6 responded in a similar manner to Tpm1, with upregulation of the PCE upon PTBP1/2 knockdown.

We noted that the PTBP1/2 regulated Atp2b4 exon 24, which is upregulated in differentiated SMCs, not only has multiple PTB-binding motifs on the upstream side, but also has 5 UAA-containing motifs (CUAA, 3 × UUAAC and UUAA) in its downstream flank, similar to the STAR-protein like motifs downstream of DCEs (Figure 4A). A minigene containing Atp2b4 exon 24 and 304 nt of each flanking intron showed increased exon inclusion in response to Ptbp1/2 knockdown, as expected (Figure 4C). Moreover, co-transfection of expression vectors for STAR proteins SLM1, TSTAR, SF1 or Sam68, but not QKI, also led to increased exon inclusion (Figure 4D), suggesting that this exon might be under dual control by PTBP and STAR proteins.

The enriched motif ACUA (*P* = 4 × 10^−4^, FDR 0.038) on the downstream side of PCEs also corresponds to an optimal QKI binding motif (49,51). QKI promotes the proliferative phenotype of VSMCs, in part by promoting skipping of Myocd exon 2a (16). PCEs in the Smtn and Myo18a genes were found to have potential QKI motifs in their downstream intron flanks. We knocked down QKI in PAC1 cells (Supplementary Figure S4A) and observed decreased inclusion of both the Smtn and Myo18a exons (Supplementary Figure S4B), consistent with QKI activation of these exons via downstream binding sites. The Myo18a exon has two perfect QKI motifs within 65 nt of the downstream intron. A Myo18a minigene showed lower inclusion upon knockdown of QKI in PAC1 cells, which was partially reversed by QKI overexpression (Supplementary Figure S4C). Moreover, single UAA to UGA mutations in each of the two QKI sites severely reduced exon inclusion, while mutation or deletion of both sites abolished inclusion (Supplementary Figure S4D). Taken together, the preceding data show that PTBP proteins repress many SMC exons and also indicate a regulatory role for QKI and possibly other STAR proteins as well.

### Concerted intron retention and other non-productive splicing in contractile cells

**Figure 5.**
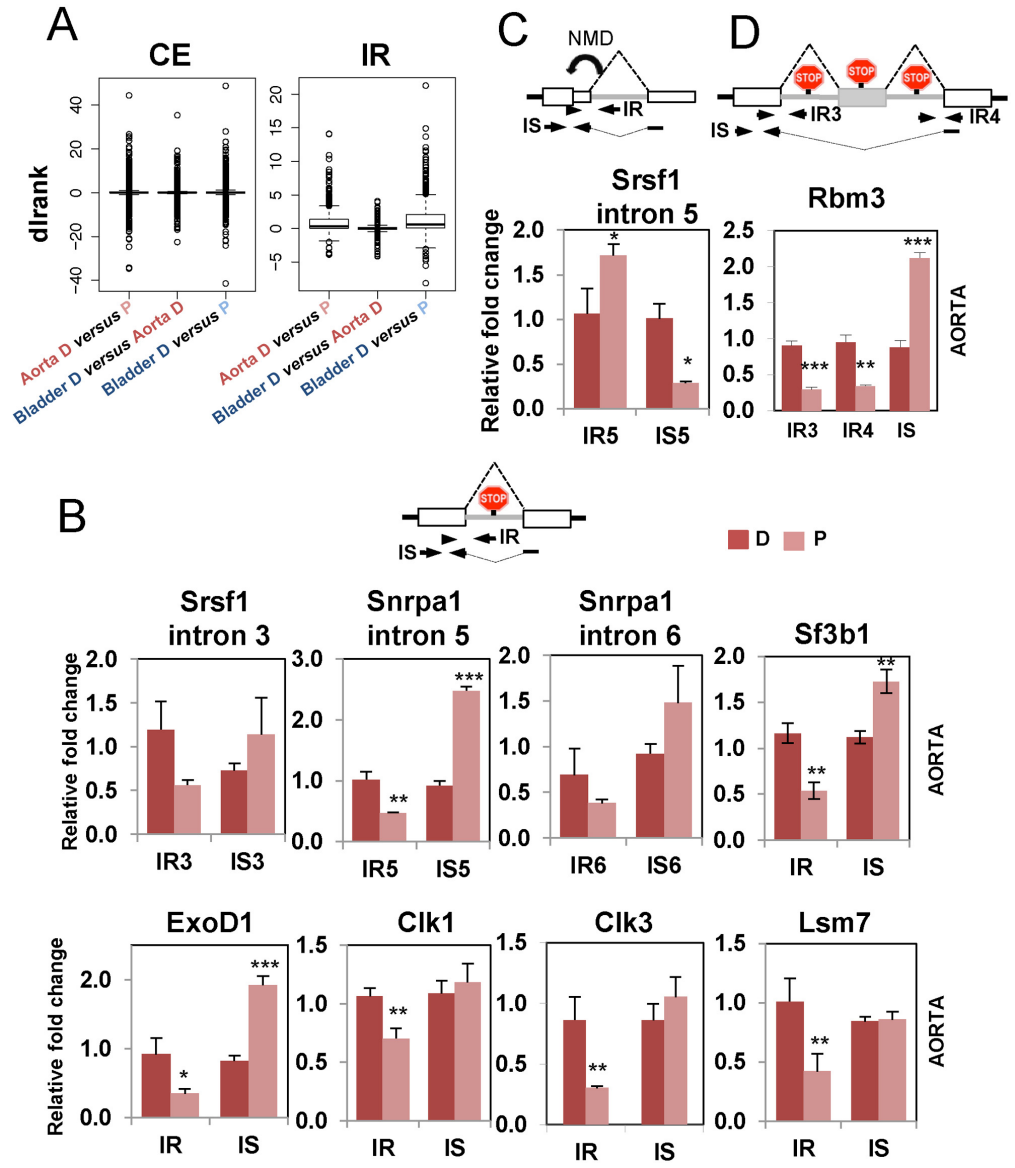
IR in splicing factor genes in differentiated SMCs. (**A**) Distribution of dIrank values of CE (left) and IR (right) events. dIrank indicates the degree of confidence in the ASPIRE predicted change in inclusion. Positive values indicate increased inclusion in differentiated cells (D versus P comparisons, aorta left, bladder right) or in differentiated bladder compared with differentiated aorta (middle). CEs show an even distribution of positive and negative dIrank values in all comparisons, while IR events are skewed toward positive values in D versus P comparisons. (**B–D**) qRT-PCR validation of IR events. Top, schematic representation of the event (in gray) with arrows showing primer position, and red ‘stop signs’ denoting premature termination codons (PTCs). Bottom, plots shown are average values of relative fold change for IR or intron spliced (IS) normalized against the geometric mean of 5 genes not changing in the microarray (see ‘Materials and Methods’ section), except for CLK1 that was normalized against its own gene expression. Dark red represents Differentiated (D) Aorta; light red Proliferative (P) Aorta. Error bars represent standard deviation of the mean (*n* = 3). (B) simple IR events with higher retention in D samples. (C) Srsf1 intron 5, showing higher retention in P samples. (D) Dual IR in Rbm3. Statistically significance was calculated using Student's *t*-test, and is shown *P < 0.05, **P < 0.01, ***P < 0.001.

In contrast to the CE events, which showed similar numbers of events upregulated in differentiated or proliferative cells, or between aorta and bladder differentiated cells (Figures 1C, 2 and 5A), IR events were highly skewed with a large excess of events with higher IR in differentiated cells (Figure 5A, positive dIrank values). While 216 introns showed higher retention in differentiated aorta, only 14 showed higher retention in proliferative aorta. Likewise in bladder, 275 introns showed higher retention in differentiated compared with only 24 for proliferative cells. In most cases IR leads to PTC insertion and downregulation of functional protein expression, either due to protein truncation, nonsense mediated decay (NMD) or RNA nuclear retention (52–57). A possible explanation for the higher levels of observed IR in differentiated cells would be global downregulation of NMD, rather than regulation of splicing. Arguing against this interpretation, 66% (143/216) of genes with higher IR in differentiated aorta were associated with decreased transcript levels (dT <1), while 86% (12/14) of genes with decreased IR had increased transcript levels. In contrast, CE splicing showed no association with transcript levels; 52% (382/735) of ‘differentiated’ CE and 49% (284/582) of ‘proliferative’ exons were associated with decreased transcript levels. Moreover, many of the RNAs with IR appear to be nuclear retained (see below), so would not be subject to NMD.

Genes with increased IR in differentiated aorta included a number of splicing factors (Srsf1, Sf3b1, Snrpa1, Wbp4, Lsm7), splicing regulatory kinases (Clk1, Clk3), 3′ end processing factors (Cstf2), the adenosine N6 methyltransferase (Mettl3) and the cold shock RNA binding protein Rbm3. Validated simple IR events are shown in Figure 5B and Supplementary Figure S5. These include intron 3 of Srsf1, which was previously shown to lead to nuclear retention of the RNA (54), introns 5 and 6 in Snrpa1 (encoding U2A′ protein), as well as introns in Sf3b1, Lsm7, Clk1, Clk3 and Exod1. In Srsf1, a second IR event (Figure 5C) showed atypical increased retention in proliferative cells. However, this intron lies within the 3′ UTR and its splicing leads to NMD (54) (Supplementary Figure S7), so this event would also lead to downregulation of protein expression in the contractile phenotype cells. Notably, the behavior of this well documented AS-NMD event, also argues against global downregulation of NMD in differentiated cells. In the case of the cold shock RNA binding protein Rbm3 two introns flanking a poison CE showed increased retention in differentiated cells, coupled with reciprocal decrease in productive splicing which involves skipping of the CE (Figure 5D).

**Figure 6.**
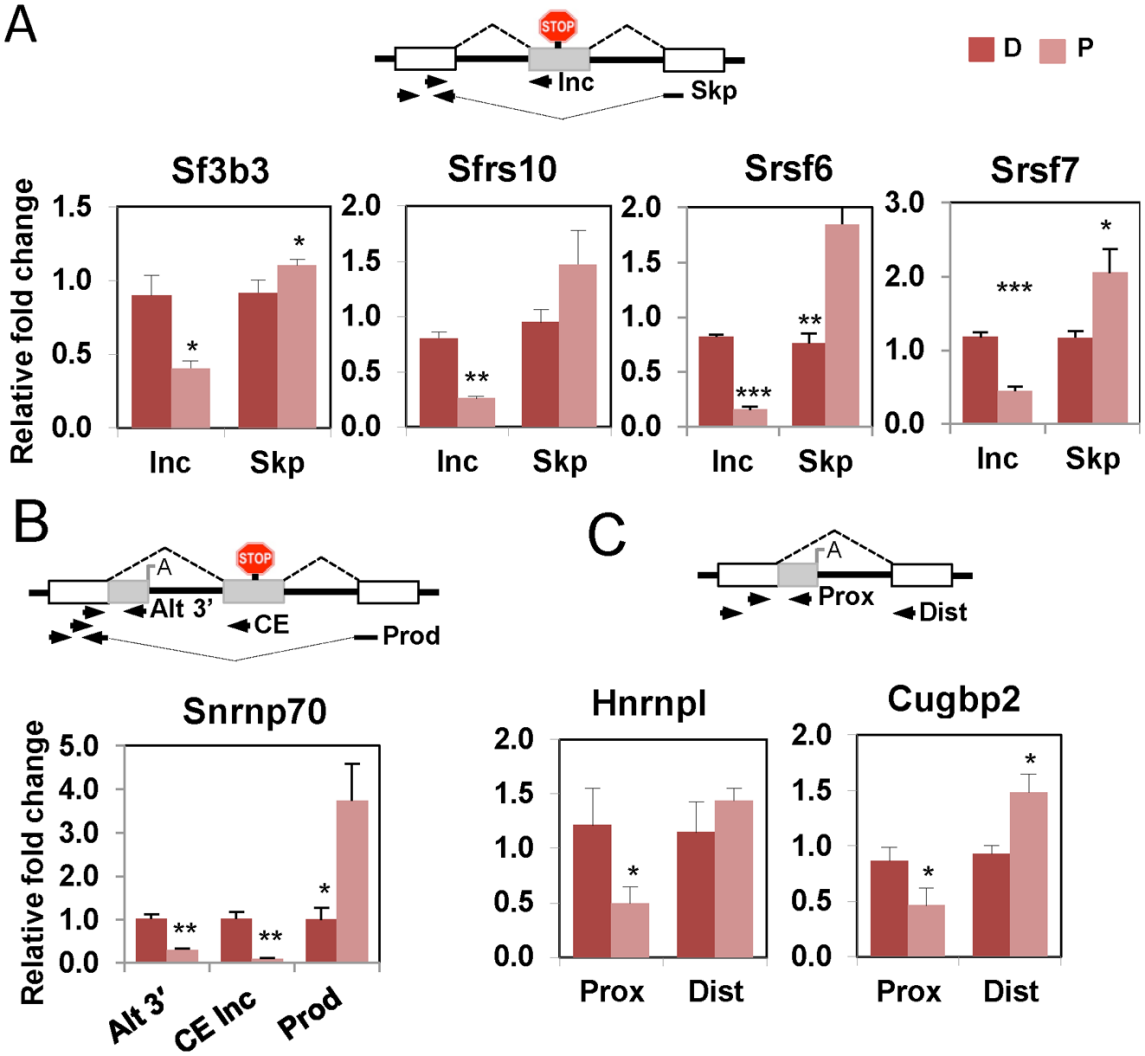
Other non-productive processing of splicing factors and regulators. (**A**) ‘Poison’ CE inclusion events. (**B**) Combined non-productive processing by internal polyA site selection and poison CE inclusion in Snrnp70. **(C)** Internal polyA site selection. Top, schematic representation of the regulated event (in gray) with arrows showing primer position and red ‘stop signs’ denoting PTCs. Bottom, histograms showing average values of relative fold change for each primer pair normalized against the geometric mean of five genes not changing in the microarray, except Sf3b3 that was normalized against its own gene expression. Dark red represent Differentiated (D) samples, light red Proliferative (P) samples. Error bars represent standard deviation of the mean (*n* = 3). Exon inclusion (Inc), exon skipping (skp), alternative 3′ end proximal (Prox), alternative 3′end distal (Dist). For Snrnp70 gene Alternative 3′ end (Alt3′), CE inclusion (CE Inc) or CE skipping (Prod). Statistically significance was calculated using Student's *t*-test, and is shown *P < 0.05, **P < 0.01, ***P < 0.001.

Re-examination of CE events revealed additional non-productive splicing events in splicing factor genes. Increased inclusion of PTC-containing ‘poison’ CEs in differentiated cells was observed for Sf3b3, Tra2β (Sfrs10), Srsf6, Srsf7 and Snrnp70 (Figure 6A, B and Supplementary Figure S6). In addition to the known poison CE in the Snrnp70 gene (58) encoding the U1 snRNP 70K protein, a second non-productive event involving an internal poly(A) addition site was also observed. Both of the non-productive splicing patterns in Snrnp70 were higher in differentiated cells, while the productive protein-coding splicing pattern increased 4-fold in the proliferative cells (Figure 6B). Finally, we also validated increased non-productive splicing of annotated ‘bleeding exon’ events in Hnrnpl and Cugbp2 in differentiated cells (Figure 6C). These two events could either be genuine alternative polyadenylation events (the Hnrnpl event has a consensus AAUAAA 15 nt from the end of the annotated exon extension) or IR, since both annotated events are followed by genomically encoded oligo A stretches.

We were unable to test whether any of the regulated non-productively spliced RNAs were substrates for NMD or nuclear retained in the tissue samples used for the array analysis. However, we were able to detect some of these events in rat PAC1 VSMCs (31). We therefore tested whether cycloheximide treatment affected the ratio of spliced products in a manner consistent with inhibition of NMD (Supplementary Figure S7A) (52), and whether any of these RNA species were preferentially retained in the nucleus (Supplementary Figure S7B). We tested the effects of cycloheximide upon the non-productive CE events and also the 3′ UTR IR event in Srsf1 (Supplementary Figure S7A). Poison CE events in Sf3b3, Srsf7, Sfrs10 (Tra2 β) and Snrnp70 all showed apparent increased exon inclusion in response to cycloheximide. Srsf1 intron 5 showed a decreased proportion of IR (consistent with stabilization of the 3′ UTR spliced product). These results are consistent with the non-productive splicing patterns of these ASEs being linked to NMD. Interestingly, we observed cycloheximide stabilization of a non-productive Snrnpa1 product with exon 6 skipped in PAC1 cells (Supplementary Figure S7A). In mouse primary samples we had observed retention of both introns flanking exon 6, but not the exon 6 skipping event.

All but one of the detected IR events were preferentially nuclear retained (Supplementary Figure S7B). The only exception was Srsf1 intron 5, the sole IR event that was upregulated in proliferative SMCs (Figure 5C) and an intron whose splicing leads to NMD. CE events in Sf3b3 and Snrpa1 showed no nuclear retention. However, both the non-productive Snrnp70 splicing events showed some nuclear retention. The poison CE was partially retained in the nucleus, while the annotated alternative polyA event was highly nuclear retained, suggesting that the detected RNA probably corresponds to IR (Supplementary Figure S7B). Of note, inclusion of the Snrnp70 poison CE results in an RNA that is both preferentially nuclear retained as well as an NMD substrate.

The retained intron in CLK1 can be post-transcriptionally spliced in response to heat or osmotic stress or upon administration of small molecule CLK inhibitors (57,59). Consistent with this we observed that addition of the CLK inhibitor TG003 (20 μM) (59) to PAC1 cells led to rapid splicing of the retained CLK1 intron, but none of the other IR events that we had detected (Supplementary Figure S7C), indicating that the SMC IR events are not all under a common mode of control with CLK1.

### Properties of smooth muscle retained introns

To investigate the properties of the regulated retained introns we compiled a set of 123 introns that were more retained in both aorta and bladder differentiated cells. These were compared with a control set of annotated retained introns that were not regulated in aorta or bladder, and also with a set of annotated constitutively spliced introns. Regulated retained introns were significantly shorter than constitutively spliced introns (*P* = 1.8 × 10^−8^), but longer than the unregulated control IR (*P* = 0.002) (Figure 3C). Consistent with this the GC content of control IR introns was higher than that of regulated IR introns (*P* = 0.0001891) or introns flanking CEs (*P* < 5.772e-16) (Figure 3D). The splice sites of regulated IR were slightly weaker than constitutive splice sites, but stronger than those of the unregulated IR events (Figure 3E), while the distance of the branch point from the 3′ exon was smaller for both regulated and unregulated IR compared to all other datasets (Figure 3H). The latter is probably related to the short length of these introns (Figure 3C).

We next examined enriched sequence motifs associated with IR events (Supplementary Figure S8). When compared to CI from the same genes, 24 3-mer motifs were found to be enriched, all but one of them in the flanking exons (Supplementary Figure S8A). In contrast, compared to unregulated IR introns, enriched motifs were only found within the intron; four motifs were enriched at the 5′ end of regulated IR and three at the 3′ end (*P* < 0.01, FDR < 0.05). The 3-mer GUG was enriched at the 3′ end of IR introns compared with both unregulated IR and CIs (Supplementary Figure S8A and B). Three GUG containing k-mers (UGUG, UUGU, UGUGC) were also enriched at the 5′ end of IR introns. Two other significant motifs at the 3′ end of regulated IR were the RNA-compete motif for KHDRBS2 (also known as SLM1) RAUAAAM and the related pentamer UUAAA, which matches the optimal UWAA site for STAR family members (49). These enriched motifs suggest possible mechanisms by which IR events may be regulated (see ‘Discussion’ section).

### Variation of splicing factors and snRNAs

**Figure 7.**
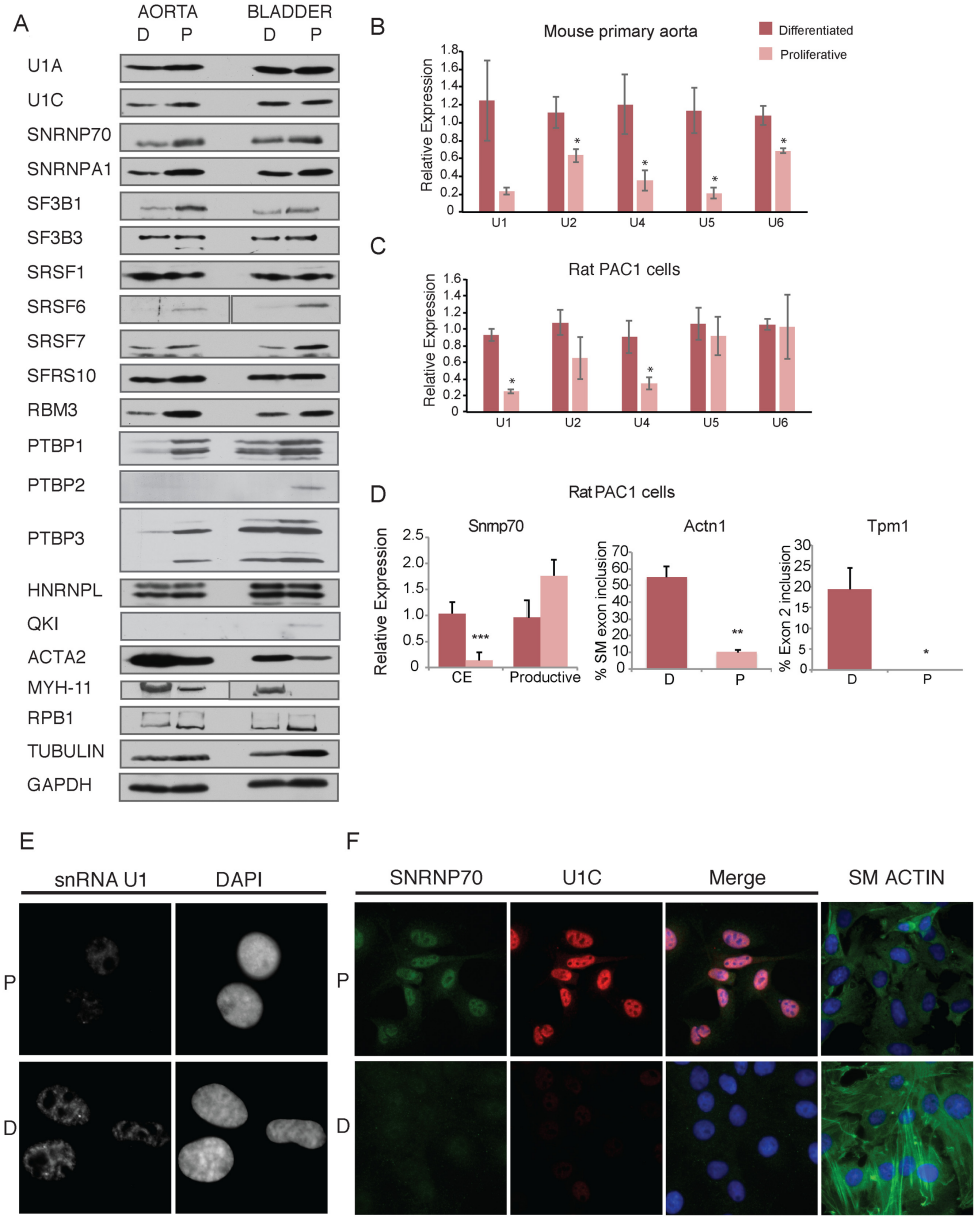
Divergent levels of U1 snRNP proteins and U1 snRNA during phenotypic modulation. (**A**) Western blots for different splicing factors and RNA binding proteins in mouse aorta and bladder samples, comparing differentiated (D) and proliferative (P) samples. Acta2 and Myh-11 antibodies are markers of smooth muscle differentiation. Rpb1, Tubulin and Gapdh are loading controls. snRNA expression levels in mouse (**B**) and rat PAC1 cells (**C**) measured by qPCR. Primers for snRNA levels were normalized against the same set of genes as in Figures 5 and 6. Error bars represent standard deviation of the mean (*n* = 3). Statistically significance was calculated using Student's *t*-test, and is shown *P < 0.05. (**D**) RT-PCR for Snrp70 (qPCR with primers as in Figure 6) and Actn1 and Tpm1 (with primers as in Figure 2) in P and D Rat PAC1 cells. Error bars represent mean and standard deviation, (*n* = 3). (**E**) Left panels: RNA-FISH for U1 snRNA in rat PAC1 D and P cells. Right panels show DAPI staining for nuclei. (**F**) Immunofluorescence in rat PAC1 cells for SNRNP70 and U1C. Sm Actin is a marker of SMC differentiation.

Taken together, the preceding data indicates widespread higher levels of non-productive splicing of various RNA splicing factors and other RNA processing and binding proteins in differentiated SMCs, accompanied in many cases by a reciprocal reduction in the levels of the productive protein-coding mRNA isoforms (e.g. Srsf1, Srsf6, Snrnp70, Snrpa1, Sf3b1, Sf3b3, Exod1, Rbm3, Cugbp2). Non-productive splicing of RNA binding proteins frequently occurs in response to high levels of the cognate protein as a result of negative feedback loops (52,60–63)). To address whether this is the case in the differentiated SMCs, we carried out western blots on protein extracted from differentiated and proliferative SMCs from mouse aorta and bladder (Figure 7A). While the levels of markers for contractile SMCs were higher in the differentiated compared to proliferative cells (Myh11, Acta2), the levels of the various splicing factors and RNA binding proteins were either higher in proliferative cells (Snrnp70, Snrnpa1, Sf3b1, Srsf6, Srsf7, Rbm3) or were not altered substantially. This suggests that in these cases unproductive splicing is not a response to high levels of the cognate proteins, but might be a contributing influence to their lower levels of expression in differentiated cells. We also looked at levels of PTB proteins, which were shown to be important in regulating many SMC ASEs (Figure 4). Notably, levels of PTBP1 protein were higher in proliferating than differentiated aorta SMCs, consistent with a role in repressing exons that are included in the contractile state. The difference in PTBP1 levels between bladder differentiated and proliferating cells was less marked. PTBP2 was not detectable, except in proliferating bladder SMCs, while PTBP3 levels changed in parallel with PTBP1.

In view of the variation in levels of core splicing factors, including snRNP proteins, we analyzed the levels of the spliceosomal snRNAs (Figure 7B). Remarkably, qRT-PCR analysis showed that levels of U1, U2, U4 and U5 snRNAs were all substantially higher in differentiated than proliferative aorta SMCs. The largest difference was observed for U1 snRNA, with 4.5-fold higher levels in differentiated cells. The elevated U1 snRNA levels contrasts with the lower levels of the U1 snRNP proteins U1 70K (Snrnp70) and U1C in differentiated aorta samples (Figure 7A). To pursue this observation further, we used cultured rat PAC1 SMCs which had been serially passaged under conditions to promote more differentiated or more proliferative phenotypes (Figure 7C–E). RT-qPCR showed 4–5 fold higher levels of U1 snRNA and 2.5-fold higher levels of U4 snRNA in differentiated compared to more proliferative PAC1 cells, while levels of U2, U5 and U6 did not differ significantly (Figure 7C). Analysis of ASEs in Snrnp70, Actn1 and Tpm1, showed changes as had been observed in the mouse primary cells; in particular Snrnp70 showed higher levels of non-productive splicing in the differentiated cells (Figure 7D). RNA-FISH images also consistently showed higher levels of U1 snRNA in differentiated compared to proliferative PAC1 cells (Figure 7E). In contrast, immunofluorescence microscopy showed levels of U1 70K (Snrnp70) to be lower in differentiated compared to proliferative PAC1 cells (Figure 7F), paralleling the changes in non-productive splicing (Figure 7C). These alterations in stoichiometry of U1 snRNP components could have important consequences for the various functions of U1 snRNP, including in regulation of AS.

## DISCUSSION

### AS reshapes the SMC proteome

Our data demonstrate that AS plays a major role in re-shaping the transcriptome and proteome of dedifferentiating SMCs. In particular, AS modulates the actin cytoskeleton, affecting genes such as Tpm1, Actn1, Cald1 and Smtn, to convert a machinery suitable for generating contraction at the tissue level to a form suitable for individual cell motility. For example in Actn1, the NM exon contributes to a functional Ca^2+^-binding EF hand, while the SM exon lacks ligands for Ca^2+^-binding so that α-actinin containing structures remain stable during Ca^2+^-induced contraction (40). We also observed AS changes affecting membrane voltage-dependent Ca^2+^ entry channels (Cacna2d1) and Ca2+ efflux pumps (Atp2b4), which are important for control of contraction and relaxation in contractile cells, and also in genes for proteins involved in protein secretion and sorting between different membrane bound organelles (Sec 61a2, Sort1). A previously unreported ASE with clear predicted functional consequences is the switch-like change from predominant skipping of Sec 61a2 exon 7 in differentiated cells to >90% inclusion in proliferative SMCs (Figure 2). Exon 7 skipping causes frameshift and insertion of a PTC resulting in an mRNA predicted to be degraded by NMD and/or encoding a protein lacking 6 of 10 transmembrane domains. Sec 61a2 is involved in ER insertion of secreted and membrane proteins, so the switch to exon 7 inclusion appears to play an important role in activating the machinery for the synthetic phenotype of SMCs in which large quantities of ECM proteins are secreted (7).

AS also affects components of integrin signaling pathways, which are important in integrating extracellular signals to control SMC phenotype (8,9), including extracellular collagens, integrins (Itga7), tyrosine kinases (Csk, Fyn) and actinin (Actn1). Mutually exclusive Itga7 exons 5 and 6, encode an extracellular linker domain. This event has been described in the context of striated muscle, with the X2 variant (exon 6) being used exclusively in adult skeletal muscle (64). In contrast, the exon 5 (X1 variant) isoform predominates in adult aorta and bladder SMCs (Figure 2 and Supplementary Figure S2). The X1 and X2 variants confer different specificities for interaction with laminins (65). Itga7 interacts with cartilage oligomeric matrix protein and promotes the contractile VSMC phenotype (66), although whether this is affected by exon 5 and 6 splicing is unclear.

### Regulation of the SMC splicing program

Analysis of CEs regulated during phenotypic modulation indicated a number of distinct features (Figures 3 and 4). They are shorter than CEs generally, and the differentiated exons had even weaker splice sites than control or proliferative CEs (Figure 3E), even allowing for the fact that CEs generally have weaker splice sites than constitutive exons (44). Differentiated exons also had larger AG dinucleotide exclusion zones (Figure 3F) and stronger polypyrimidine tracts within the AGEZ (Figure 3G), which could mean that their branch point/polypyrimidine tract elements are strong, despite the predicted weak 3′ss, which is based only on elements within 20 nt of the 3′ss (45). Long AGEZs are also characteristic of PTB repressed exons (27), and differentiated exons showed significant enrichment on their upstream intronic flank of PTBP1 binding motifs (Figure 4A), a location where PTB binding causes exon skipping (27,48). Indeed, knockdown of PTBP1 in PAC1 cells led to increased use of differentiated CEs in Dock7, Atp2b4, Dst, Pkm and Actn1 (Figure 4B). This suggests that PTB commonly acts to repress SMC-specific exons outside of differentiated cells, as has been shown for the SM exon of Actn1 (14,15). Consistent with this, western blots indicated that PTBP1 levels were lower in aorta and bladder differentiated cells than in cultured cells (Figure 7). Nevertheless, knockdown of PTBP1/2 in PAC1 cells was not sufficient to switch splicing patterns fully to the levels of exon inclusion seen in mouse tissues (Figures 2 and 4), suggesting that other mechanisms might be involved in countering repression by PTBP1. Tpm1 exon 3 is an atypical case where PTBP1 acts to repress an exon that is used in the non-differentiated cells, allowing use of the mutually exclusive partner exon 2 (21). Itga7 exons 5 and 6 responded to PTBP1/2 knockdown in a similar fashion, with decreased use of the differentiated exon 5 and increased inclusion of mutually exclusive exon 6 (Figure 4B). Consistent with the suggestion of shared regulatory mechanisms, Itga7 exons 5 and 6 have a very similar splice site arrangement to Tpm1 exons 2 and 3, with mutually exclusive behavior predicted to arise from proximity of the exon 6 branch point to exon 5. A key question for the regulation of these two exon pairs is how PTBP1 repression can be enhanced in differentiated cells while its activity on the majority of exons is diminished.

Motif enrichments also suggested possible roles of QKI or other STAR proteins (Figure 4). Indeed, QKI regulates an overlapping set of exons with PTB in striated muscle cells (24), and represses smooth muscle specific splicing of myocardin exon 2a (16) (Supplementary Figure S4). QKI-like motifs were enriched downstream of differentiated exons, a location consistent with activation by Quaking (24). However, Quaking levels increase in dedifferentiated mouse and human VSMCs (16), so the role of Quaking in regulating DCEs is unclear. The related motif, ACUA, is significantly enriched downstream of proliferating exons (Figure 4). The decreased use of Smtn and Myo18a PCEs in response to QKI knockdown (Supplementary Figure S4) is consistent with QKI activation of some PCEs.

Analysis of regulated IR events (Figure 3 and Supplementary Figure S8) provided some hints about how they might be regulated. The observation that regulated IR events have stronger splice sites than unregulated IR (Figure 3) is consistent with analysis of IR programs in differentiating erythroblasts, and suggests the involvement of regulatory factors in modulating IR (67). For example, retention of 3′ terminal introns downregulates expression of pre-synaptic proteins in non-neuronal cells and is promoted by PTBP1 (68), while Sam68 promotes retention of an Srsf1 intron (69). The SMC regulated IR introns are enriched for GUG-containing motifs (Supplementary Figure S8). Notably, three of the seven motifs enriched downstream of proliferative CEs also contained a GUG motif (UCGUG, AUGUG, UGUGU, Figure 4), suggesting that GUG-containing motifs may be important in modulating the use of 5′ss between differentiated and proliferative cells, both in the context of IR and CEs. Whether they act to downregulate splicing in differentiated cells and/or to activate in proliferating cells is, as yet, unclear. A number of RNA binding proteins are known to bind GU-rich motifs including CELF proteins (36,70), TDP43 (71) and ESRP1 and 2 (72). In addition, two UA rich motifs were enriched at the 3′ end of regulated retained introns, including the RNA-compete motif for KHDRBS2 (also known as Sam68 Like Mammalian protein 1). Future work will aim to determine the pathways that lead to concerted IR and other non-productive splicing in differentiated SMCs, and whether these converge upon Sam68-like or GUG-binding proteins.

Notably, the proteins that we and others have identified to be involved in regulating SMC AS, including PTBP1 and QKI are expressed at higher levels in the non-differentiated phenotype (Supplementary Figures S1 and 7, (16)). The under-representation of RNA binding proteins (RBPs) from genes that are transcriptionally regulated between differentiated or de-differentiated cells was initially surprising as we expect RBPs to play an important role in orchestrating changes in AS. However, RBPs can be regulated by translation, post-translational modifications and localization. In addition, the small number of RBPs that are regulated at the transcript level become interesting candidate regulators, especially if their genes are associated with tissue-specific superenhancers (73), indicative of proteins with critical roles in identity of the differentiated phenotype. The tissue samples that we used here were useful in order to profile the transcriptomes of fully differentiated SMCs as found *in vivo*. However, this system had the attendant difficulty of experimental follow up. In the future we aim to analyze SMC AS programs and mechanisms in an experimentally amenable system via controlled differentiation from embryonic stem cells (74,75).

### Non-productive splicing of splicing factors

Our data highlighted a concerted set of changes in non-productive splicing within genes for splicing factors and other post-transcriptional regulators, all of which are predicted to downregulate protein expression in the differentiated contractile cells. The non-productive splicing events affected SR family members (Srsf1, Srsf6, Srsf7, Sfrs10/Tra2 β), components of U1 (U1 70K/Snrnp70) and U2 snRNP (Snrpa1, Sf3b1, Sf3b3), but not of U4/5/6 snRNPs. The events included IR, ‘poison’ CE inclusion, and alternative 3′ end formation (Figures 5 and 6). As well as simple cases of individual IR, we also saw cases of dual IR flanking CEs which can lead to inclusion of PTCs when included (e.g. Rbm3) or when skipped (e.g. Snrpa1, Figure 5B and D). The non-productive splicing patterns are all expected to lead to downregulation of gene expression, either by NMD of the PTC containing products (52) or by nuclear retention of the unspliced RNAs (57,68). We were unable to address which pathways were used in the differentiated mouse tissue samples used for the array analysis. However, using PAC1 cells we showed that some of the poison CE events were responsive to cycloheximide, consistent with degradation by the translation-dependent NMD pathway, while most of the IR events were preferentially nuclear retained (Supplementary Figure S7). Strikingly, the one case of IR that increased in proliferative cells was a 3′ UTR intron in Srsf1 (Figure 5C). But in this case, productive splicing leads to a non-productive mRNA which is a substrate for NMD (54,69) (Supplementary Figure S6); so this event would also contribute to upregulation of protein expression in the proliferative cells.

The scope of regulated IR and its role in controlling gene expression has become increasingly apparent over the past 2 years (55–57,76–78). IR was observed to be an intrinsic part of the granulocyte differentiation program (77), of the heat shock response (55), and characteristic of many cancers (78). Moreover, a subset of apparent IR events, referred to as detained introns, were shown to be retained in the nucleus and subsequently spliced in response to specific signaling pathways (57). A series of dynamically regulated non-productive splicing events (79), including IR (67), was also observed in erythroid cell differentiation and included many of the same events affecting splicing factor genes that we observed here. IR was shown to be accompanied by nuclear retention of the mRNAs (67). Our observations show that regulated non-productive splicing of splicing factor genes is not restricted to differentiated hematopoietic cells. Indeed, using ASPIRE to analyze published array data using for differentiating mouse myogenic C2C12 cells (24), we observe many of the same non-productive splicing patterns to be upregulated in differentiated C2C12 myotubes. In most cases the array predicted changes were more modest in C2C12 cells than aorta or bladder SMCs, possibly reflecting the higher degree of quiescence of differentiated cells in tissue compared with *in vitro* differentiated C2C12 cells. Taken together, the available data suggest that concerted downregulation of post-transcriptional regulators by a range of non-productive splicing events might occur in many quiescent non-proliferating cell-types. Our data shows that the non-productive splicing ‘sub-program’ is not exclusively associated with terminally differentiating cells like erythroblasts (67), granulocytes (77) and that it can be reversed in cells with phenotypic plasticity such as dedifferentiating SMCs.

Many non-productive splicing events within the genes for splicing factors and other RBPs are able to operate as negative feedback loops that act to prevent over-expression of the cognate factor, often via NMD (52,60–63). This is not the case in SMCs, where we observed lower levels of the corresponding protein accompanying the non-productive splicing in differentiated cells (Figure 7). This suggests that in this case the non-productive splicing responds to signaling that actively downregulates the steady state level of proteins, possibly re-setting the level maintained by negative feedback (73).

### The core splicing machinery in transcriptome regulation

Experimental knockdown of core splicing factors has the primary effect of altering AS splicing patterns, rather than globally disrupting splicing (80,81). Moreover, physiological alteration in the levels of the U2 snRNP protein Sf3b1 affects specific ASEs with clear functional consequences (82). We observed reductions in the levels of U1 (U1 70K) and U2 snRNP (Snrpa1, Sf3b1) proteins in aorta and bladder differentiated cells (Figure 7A), all of which were seen to affect multiple ASEs upon knockdown (80). Strikingly, the levels of the cognate snRNAs did not change in parallel. This was most obvious for U1 snRNP where the levels of U1 snRNA and U1 snRNP proteins changed in opposite directions between differentiated and proliferative cells (Figure 7B–D). We cannot currently say whether the differentiated cells have an excess of U1 snRNA over U1 70K protein (encoded by Snrnp70), or whether proliferative cells have an excess of U1 70K over U1 snRNA. The non-productive splicing event in Snrnp70 can be promoted by a negative feedback loop involving U1 70K and U1C proteins (58). In the SMCs, this splicing event is not primarily driven by the feedback loop, as the non-productive splice occurs in the differentiated state with lower U1 70K protein and higher U1 snRNA, which would be expected to promote the productive splicing pattern (58). It is an intriguing possibility that in the differentiated SMCs there is a substantial population of sub-stoichiometric U1 snRNP lacking U1 70K and U1C proteins, and that this heterogeneity in U1 snRNP composition might play a role in regulating AS. Indeed, an implication of the Snrnp70 feedback loop is that productive Snrnp70 splicing uses a sub-stoichiometric U1 snRNP lacking U1 70K (58). It is an interesting possibility that the differentiated cell CEs with particularly weak 5′ss (Figure 3) might be preferentially activated by sub-stoichiometric U1 snRNP particles in a similar way, possibly helping to activate the exons at the same time that repression by PTBP1 is relieved. U1 snRNP also has other roles in gene expression, including in the suppression of promoter proximal polyA sites, a process that involves base-pairing of U1 snRNA with target RNAs (83,84), and may also require U1 70K protein (85). Widespread shortening of 3′ UTRs via use of proximal polyA sites occurs during cellular activation and proliferation (86,87). It is therefore an interesting possibility that changes in U1 snRNA levels and snRNP composition during SMC phenotypic modulation could also affect polyA site selection and 3′ UTR length. Our detection of U1 snRNA was based on the sequence of the canonical U1 sequence. However, it is possible that expression of U1 variants might also play a role in differentiated SMCs (88). Finally, the imbalance between U1 snRNA and U1 70K levels also has implications for snRNP assembly, in light of the recent demonstration that U1 70K acts as a U1-specific snRNP assembly factor, favoring assembly of U1 at the expense of the other spliceosomal snRNAs (89).

Models for regulation of AS have often posited that the balance of positively and negatively acting regulators can determine splicing outcomes. Our findings demonstrate that this concept should be extended to include the expression of core splicing machinery, and possibly heterogeneous snRNP composition as additional influences upon cell-specific splicing.

## ACCESSION NUMBER

Data have been deposited in Array Express under accession number E-MTAB-4841.

## Supplementary Material

SUPPLEMENTARY DATA
