# Supplementary material for: The alternative splicing program of differentiated smooth muscle cells involves concerted non-productive splicing of post-transcriptional regulators
Source: Nucleic Acids Res. 2016 Jun 17;44(18):8933–50. doi: 10.1093/nar/gkw560 (PMC5062968; doi:10.1093/nar/gkw560)
Supplement: SUPPLEMENTARY DATA [file supp_44_18_8933__index.html]

The alternative splicing program of differentiated smooth muscle cells involves concerted non-productive splicing of post-transcriptional regulators — The alternative splicing program of differentiated smooth muscle cells involves concerted non-productive splicing of post-transcriptional regulators — SUPPLEMENTARY DATA 

# The alternative splicing program of differentiated smooth muscle cells involves concerted non-productive splicing of post-transcriptional regulators

## SUPPLEMENTARY DATA

- SUPPLEMENTARY DATA
- SUPPLEMENTARY DATA
- SUPPLEMENTARY DATA
- SUPPLEMENTARY DATA
- SUPPLEMENTARY DATA
