# Supplementary material for: The alternative splicing program of differentiated smooth muscle cells involves concerted non-productive splicing of post-transcriptional regulators
Source: Nucleic Acids Res. 2016 Jun 17;44(18):8933–50. doi: 10.1093/nar/gkw560 (PMC5062968; doi:10.1093/nar/gkw560)
Supplement: SUPPLEMENTARY DATA [file supp_gkw560_nar-01411-a-2016-File009.pdf]

Supplementary Figures 1-8

The alternative splicing program of differentiated smooth muscle cells involves concerted non-productive splicing of post-transcriptional regulators

Miriam Llorian, Clare Gooding, Nicolas Bellora, Martina Hallegger, Adrian Buckroyd, Xiao Wang, Dipen Rajgor, Melis Kayikci, Jack Feltham, Jernej Ule, Eduardo Eyras, Christopher WJ Smith

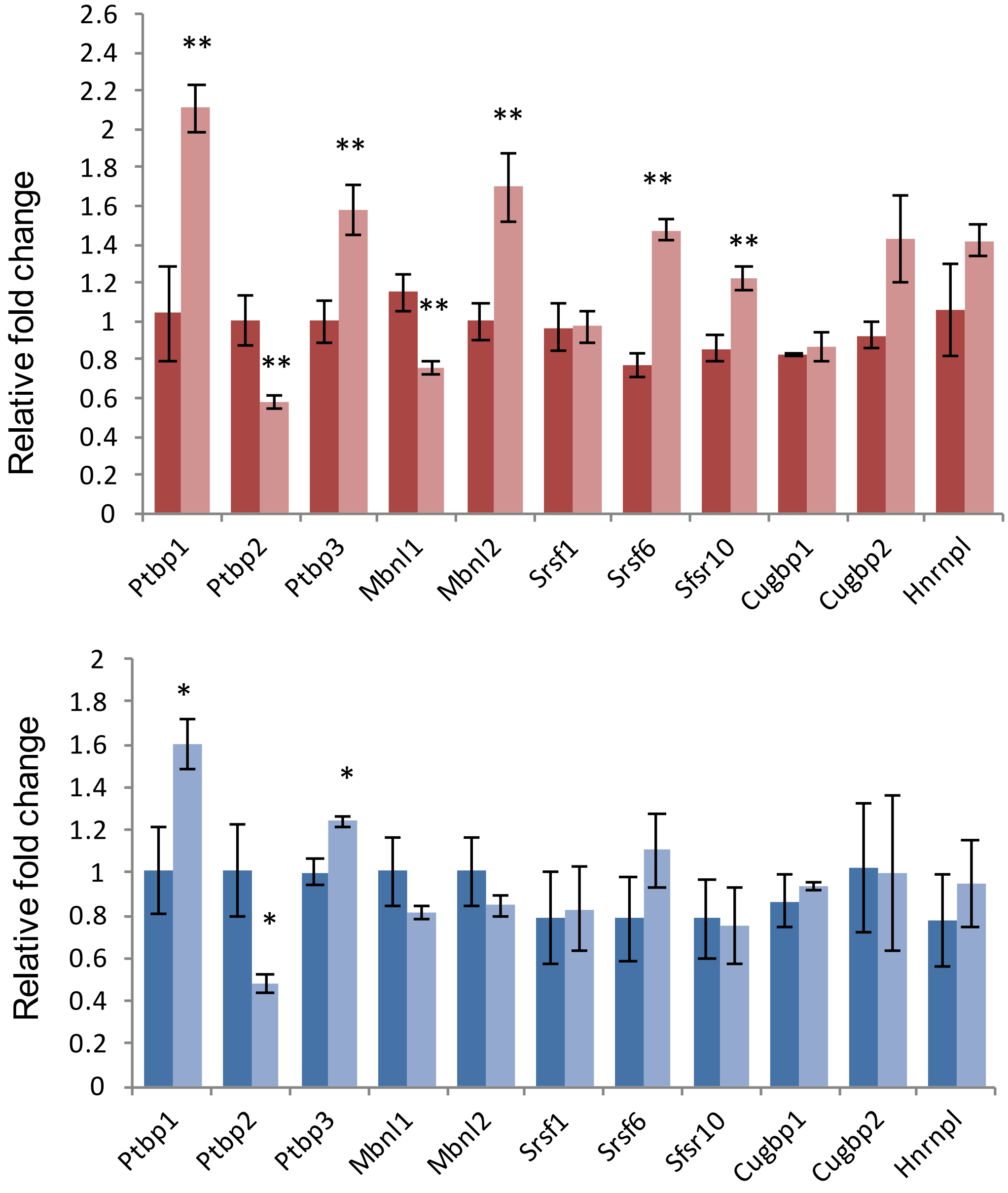

Supplementary Figure 1: Relative expression levels of RNA binding proteins and splicing factors in mouse aorta and bladder samples during phenotypic modulation. Expression levels were measured by qRT-PCR between differentiated (dark) and proliferative (light) aorta (red) and bladder (blue) samples, and values were normalized against the geometric mean of 5 genes not changing in the microarray (see methods). Values shown are mean and standard deviation of the mean (n=3). Statistically significant changes (Student's T-test) are marked * $p < 0.05$, ** $p < 0.01$.

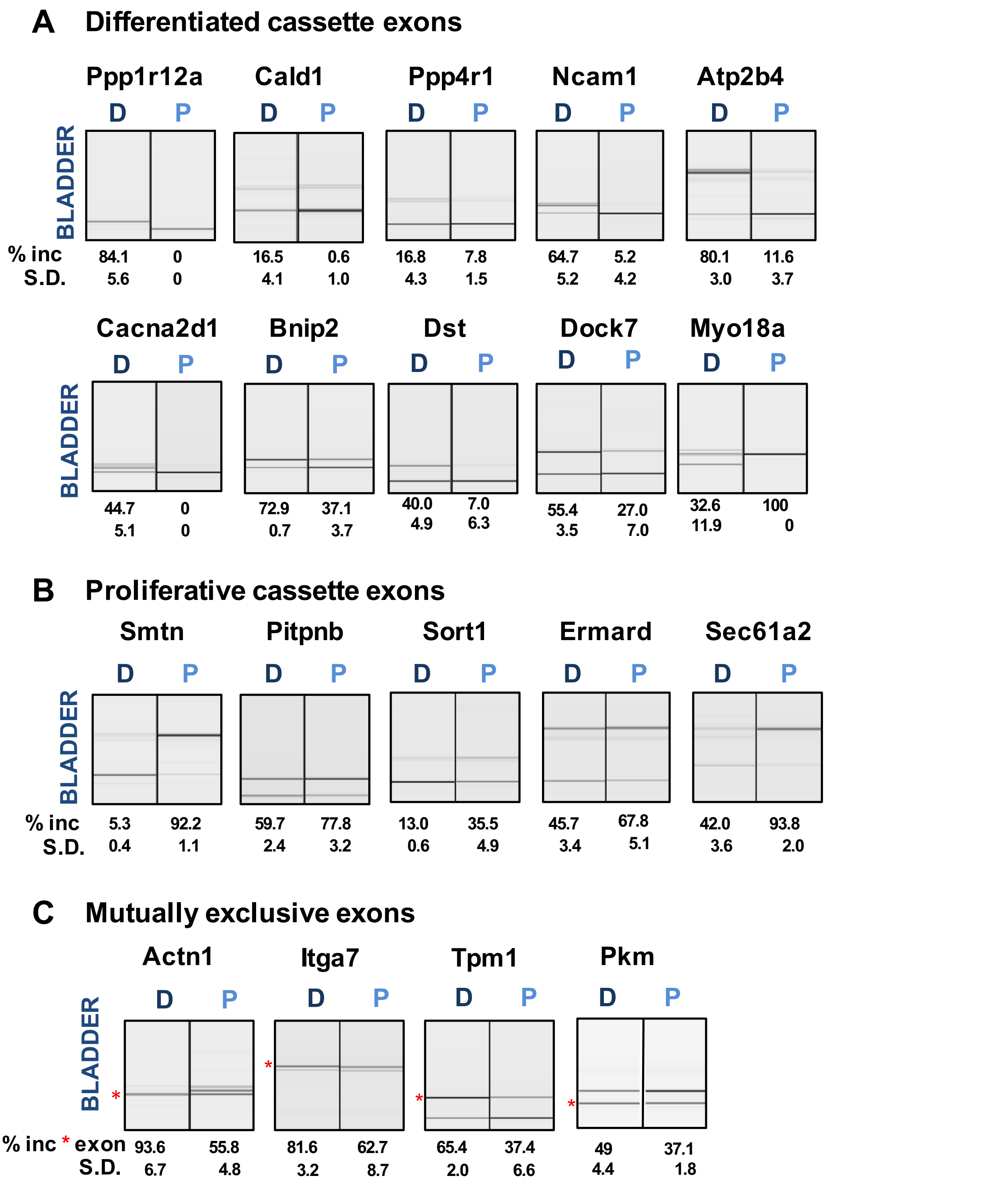

Supplementary Figure 2: Cassette exons changing during phenotypic modulation in mouse bladder. Validation of changes in exon inclusion during mouse bladder phenotypic modulation determined by RT-PCR and quantified using QIAxcel. Values shown are mean and standard deviation of the mean (n=3) for A) exons more included in differentiated (D) samples, B) exons more included in proliferative (P) samples, C) Mutually exclusive exons (the red asterisk denotes the product containing the differentiated SM specific exon)

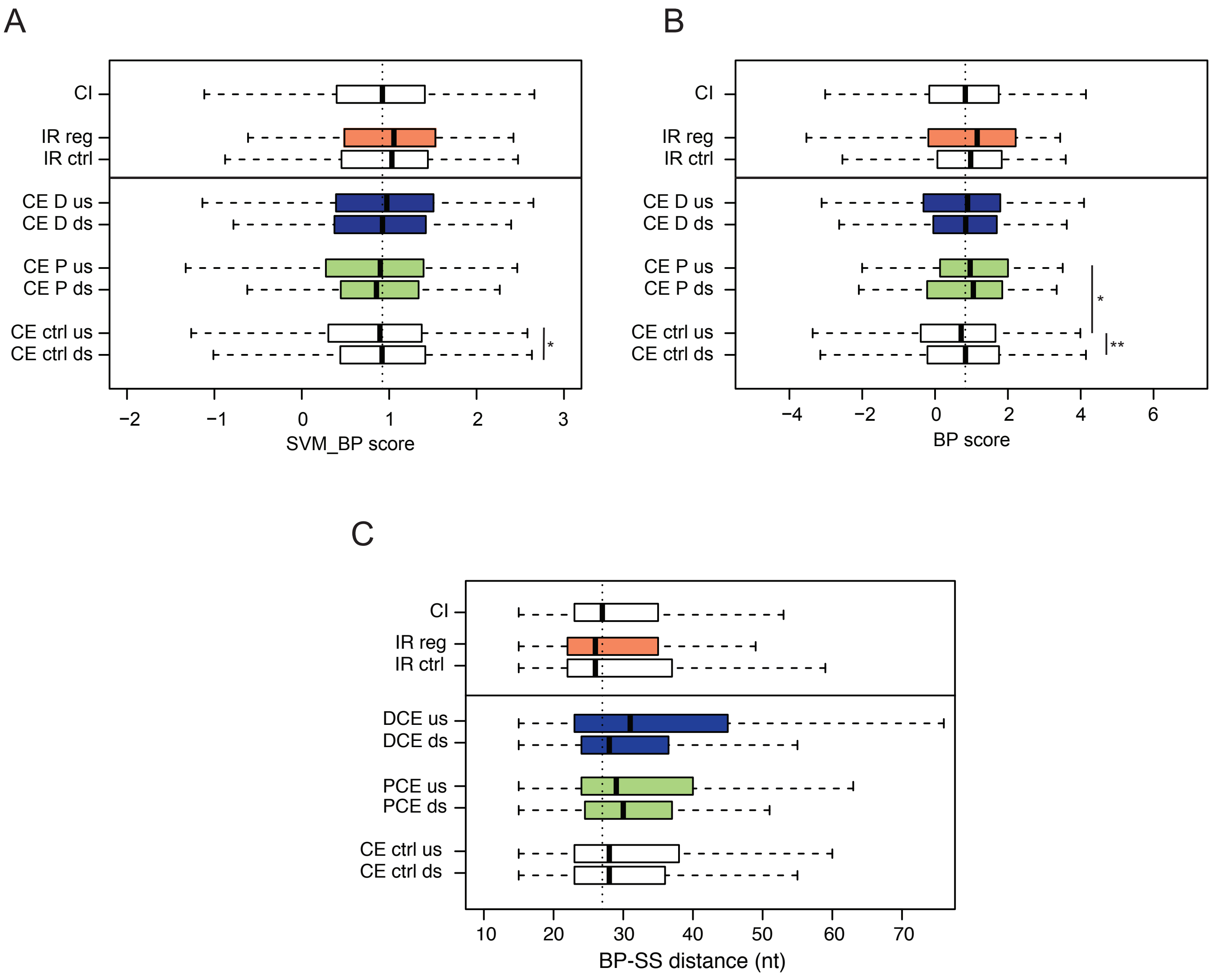

Supplementary Figure 3.
Distribution of best branch point scores in same data sets as in Figure 3. A) SVM_BP score is based on a number of features including the branch point sequence and polypyrimidine tract, while B) BP score is based only the 9nt sequence of the branch point. Highlighted statistically significant differences based in pairwise comparison Wilcoxon test.
* p-value ≤ 0.05, ** p-value ≤ 0.01. See values for other comparisons below.
C) Distance between highest scoring SVM-BP and cognate 3' splice site.

SVM_BP score
wilcox.test()$p.value :

CE D ds,CTRL ds 0.790
CE P ds,CTRL ds 0.618
CE D ds,CE P ds 0.801

CE D us,CTRL us 0.094
CE P us,CTRL us 0.640
CE P us,CE D us 0.539

CE D us,CE D ds 0.445
CE P us,CE P ds 0.855
CTRL us,CTRL ds 0.009

BP score
wilcox.test()$p.value :

CE D ds,CTRL ds 0.855
CE P ds,CTRL ds 0.393
CE D ds,CE P ds 0.409

CE D us,CTRL us 0.191
CE P us,CTRL us 0.021
CE P us,CE D us 0.269

CE D us,CE D ds 0.752
CE P us,CE P ds 0.583
CTRL us,CTRL ds 0.002

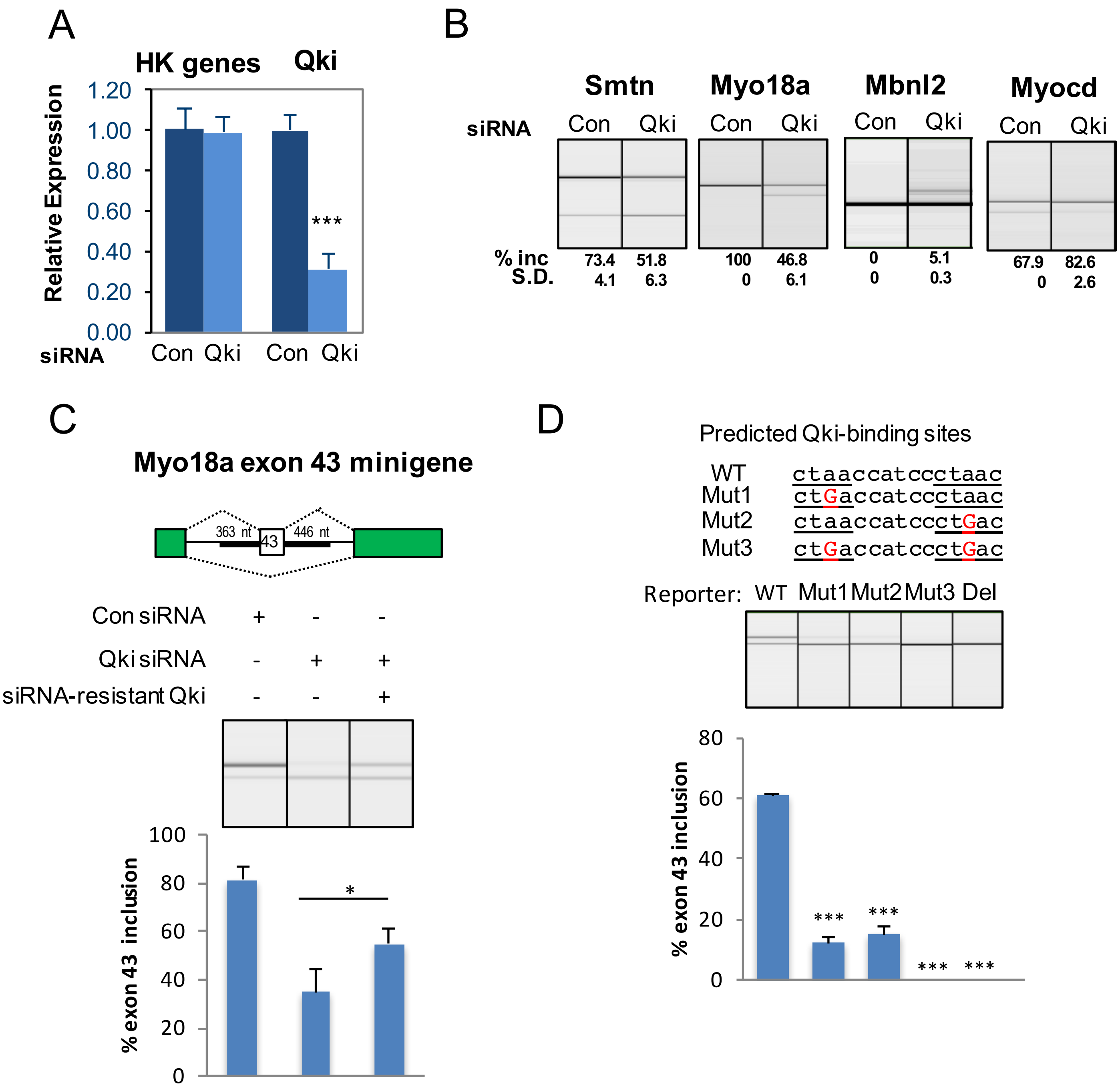

Supplementary Figure 4. Quaking regulates exon inclusion of Myo18a exon 43 in rat PAC1 cells.
A) Efficiency of Qki knockdown (KD) on PAC1 cells measured by qPCR (mean ± standard deviation, n=3). Qki expression values normalized to geometric mean of housekeeping genes.
B) Effect of Qki KD on several ASE measured by RT-PCR and quantified using QIAxcel (n=3).
C) Schematic of Myo18a minigene with exon 43, 363 nt of upstream and 446 nt of downstream intron. Effect of Qki knockdown and complementation with siRNA resistant Qki on Myo18a exon minigene measured by RT-PCR and quantified using QIAxcel.
D) Effect of point mutations and deletion of a putative Qki binding site located 45-59 nt downstream of Myo18a exon. All data shown is (mean and standard deviation, n=3). Statistically significant values were calculated using T-test, and asterisks represent: * P<0.05, ** P <0.01, *** P<0.001.

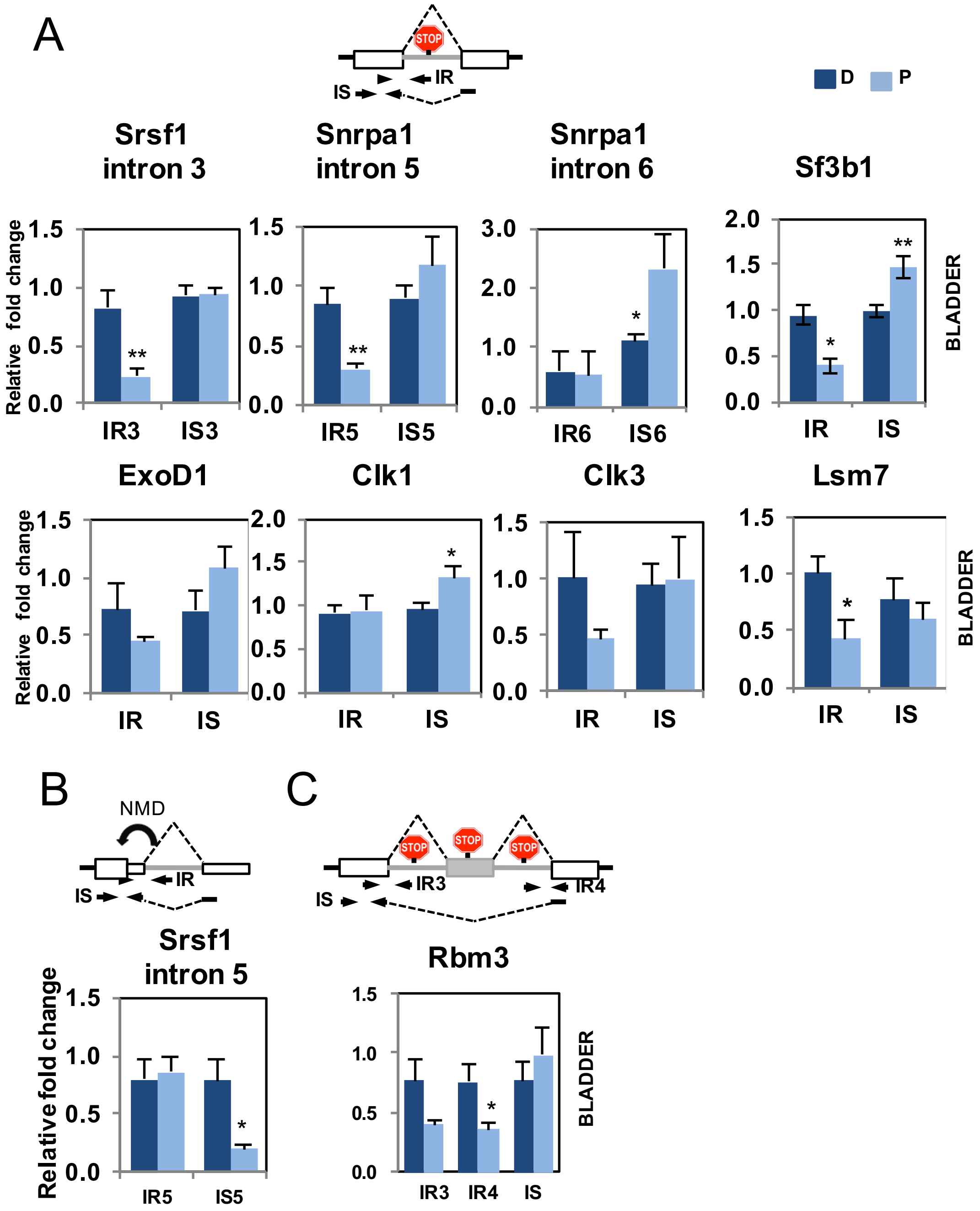

Supplementary Figure 5: Intron Retention (IR) events in RNA Binding Proteins are more included in differentiated samples (D) during bladder phenotypic modulation. Changes in IR were validated by qPCR using primers indicated in figure and normalized by geometric mean of housekeeping genes (as indicated in methods). Data shown represents mean and standard deviation of the mean (n=3). Statistically significant events (Student's T –test) are marked * P< 0.05, ** P< 0.01, *** P < 0.001.

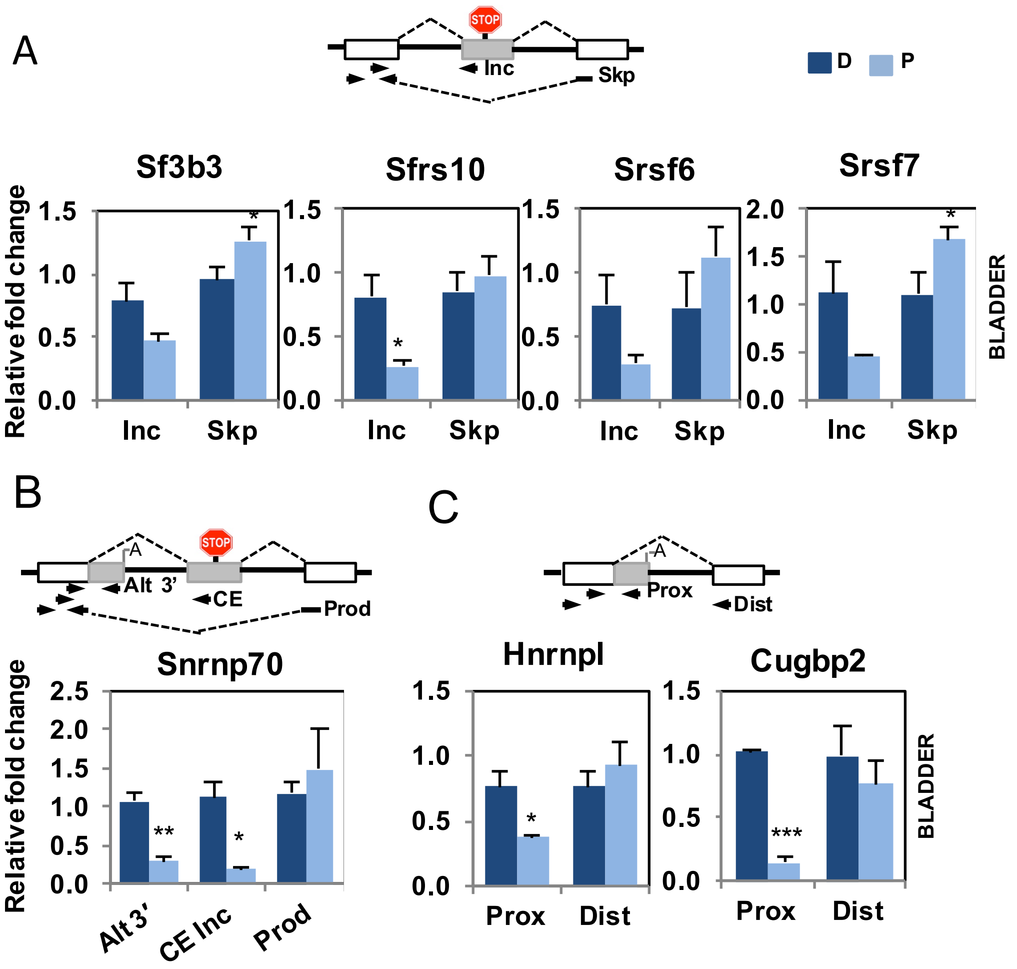

Supplementary Figure 6. Upregulation of non-productive cassette exon and alternative 3' end splicing events in genes for splicing factors and RNA Binding Proteins in bladder differentiated samples (D) during bladder phenotypic modulation.

Changes in CE or alternative 3' ends were validated by qPCR using primers indicated in figure and normalized by geometric mean of housekeeping genes (as indicated in methods).

A) Poison cassette exon events, B) Combined poison cassette exon or alternative 3' end in Snrnp70, C) Alternative 3' end events. Data shown are mean and standard deviation (n=3). Statistically significant events (Student's T –test) are marked * P< 0.05, ** P< 0.01, *** P < 0.001.

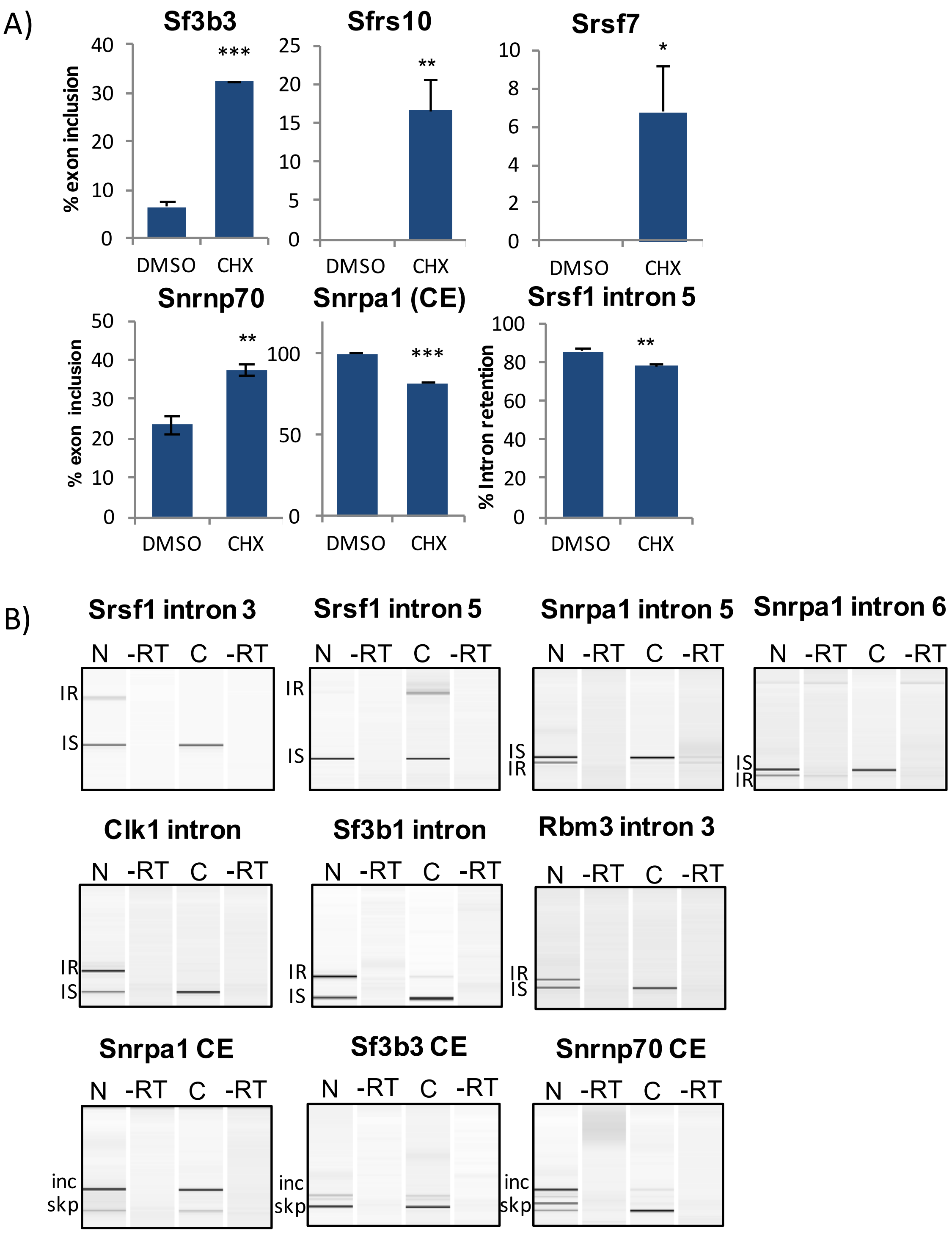
A)
Sf3b3
% exon inclusion
40
30
20
10
0
***
DMSO
CHX
Sfrs10
25
20
15
10
5
0
**
DMSO
CHX
Srsf7
10
8
6
4
2
0
*
DMSO
CHX
Snrnp70
% exon inclusion
50
40
30
20
10
0
**
DMSO
CHX
Snrpa1 (CE)
100
50
0
***
DMSO
CHX
Srsf1 intron 5
% Intron retention
100
80
60
40
20
0
**
DMSO
CHX
B)
Srsf1 intron 3
N -RT C -RT
IR
IS
Srsf1 intron 5
N -RT C -RT
IR
IS
Snrpa1 intron 5
N -RT C -RT
IS
IR
Snrpa1 intron 6
N -RT C -RT
IS
IR
Clk1 intron
N -RT C -RT
IR
IS
Sf3b1 intron
N -RT C -RT
IR
IS
Rbm3 intron 3
N -RT C -RT
IR
IS
Snrpa1 CE
N -RT C -RT
inc
skp
Sf3b3 CE
N -RT C -RT
inc
skp
Snrnp70 CE
N -RT C -RT
inc
skp

C)

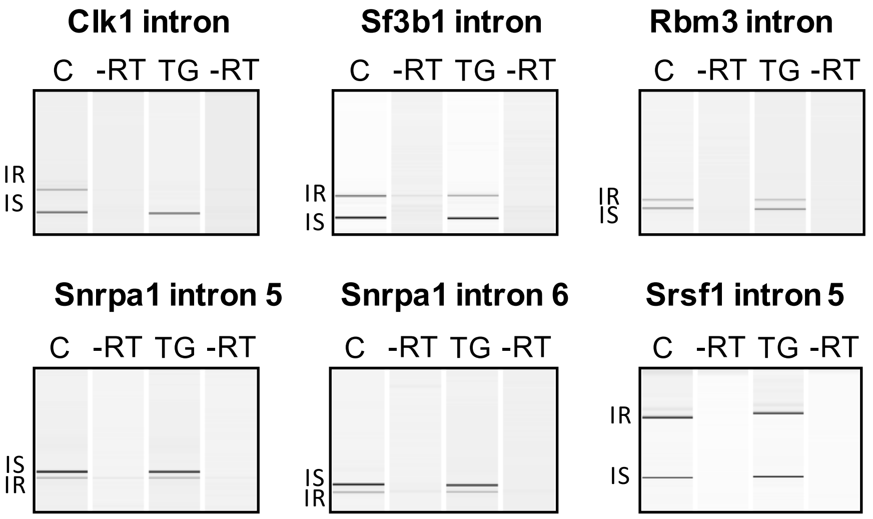

Supplementary Figure 7. Nuclear retention and cycloheximide sensitivity of non-productive ASEs in PAC1 cells

A) Rat PAC1 cells were treated with 10 ug/ml of cycloheximide for 8hrs. RNA was extracted and ASE analyzed by PCR and QIAxcelData shown is representative of 3 independent experiments. Error bars represent standard deviation of the mean (n=3). Statistically significant events (Student's T-test) is marked * p< 0.05, ** p<0.01, *** p<0.001. **B)** Subcellular fractionation. RNA was extracted from nuclear (N) and cytoplasmic (C) fractions of PAC1 cells, and the subcellular distribution of interesting ASE monitored by RT-PCR and QIAxcel. Data shown is representative of at least 4 independent fractionations. **C)** Rat PAC1 cells were treated with 20 µM of the CLK inhibitor TG003 for 2hrs. RNA was extracted and ASE analyzed by PCR and QIAxcel. C refers to control cells, PAC1 cells treated with DMSO and TG refers to PAC1 cells treated with TG003. Treatment was carried out in 3 independent experiments.

A

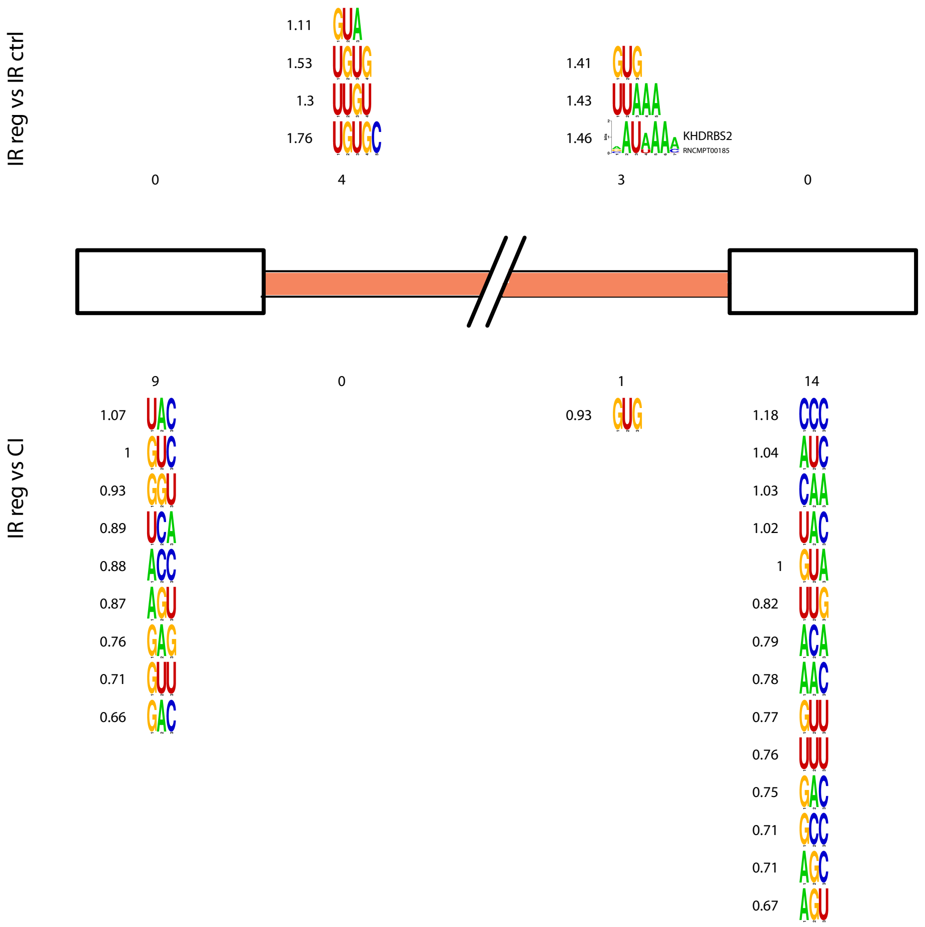

B

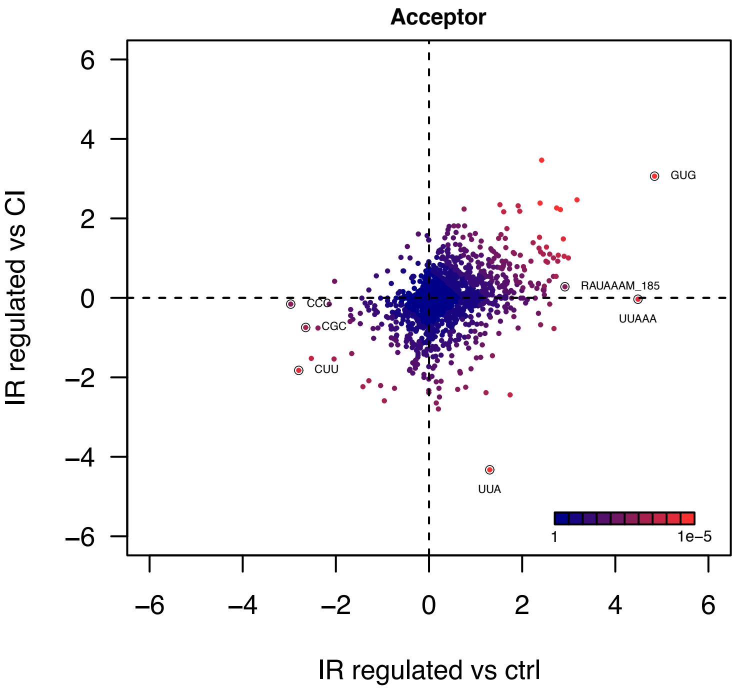

Supplementary Figure 8. Sequence motifs associated with smooth muscle cell intron retention events.
A) k-mer and RNA-compete motifs enriched in the indicated regions associated with smooth muscle regulated IR events. All motifs were significantly enriched ($p < 0.01$, FDR < 0.05). Numbers adjacent to motifs indicate log2 fold enrichment. Top: motifs enriched in comparison with unregulated annotated intron retention events. Bottom: motifs enriched in comparison with a set of constitutive introns from the same genes.
B) 2D scatter plot for the intron 3´ end. Axes indicate –lg P-value of enrichment compared to the two reference intron data-sets. Significantly enriched and depleted motifs are indicated.
